# Review of the Arabian *Crematogaster* Lund (Hymenoptera, Formicidae), synoptic list, distribution, and description of two new species from Oman and Saudi Arabia

**DOI:** 10.3897/zookeys.898.37531

**Published:** 2019-12-10

**Authors:** Mostafa R. Sharaf, Abdulrahman S. Aldawood, Francisco Hita Garcia

**Affiliations:** 1 Department of Plant Protection, College of Food and Agriculture Sciences, King Saud University, Riyadh, Kingdom of Saudi Arabia King Saud University Riyadh Saudi Arabia; 2 Division of Invertebrate Zoology, American Museum of Natural History, New York, USA American Museum of Natural History New York United States of America; 3 Biodiversity and Biocomplexity Unit, Okinawa Institute of Science and Technology Graduate University, Onna-son, Okinawa, Japan Graduate University Onna-son Japan

**Keywords:** Arabian Peninsula, Asir Mountains, Dhofar Governorate, Middle East, new records, new species, new status, new synonymy, Qatar, taxonomy

## Abstract

The genus *Crematogaster* is one of the most species-rich and widespread groups of ants. Despite their often-high local abundance and important ecological interactions, the taxonomy of the genus is fragmentary and in great need of modern revisionary studies. As a first step towards a revision for the Arabian fauna of *Crematogaster*, a review of all known species with synoptic species accounts is provided. Seventeen species are recognized and illustrated from the Arabian Peninsula, of which two new species are described: *C. jacindae* Sharaf & Hita Garcia, **sp. nov.** from the Dhofar Governorate, Oman, and *C. gryllsi* Sharaf & Hita Garcia, **sp. nov.** from the Kingdom of Saudi Arabia (KSA) based on the worker caste. *Crematogaster jacindae***sp. nov.** is easily separated from the remainder of the Arabian *Crematogaster* fauna due to its complete lack of propodeal spines, slit-shaped propodeal spiracles, and its distinct bicoloration, whereas *C. gryllsi***sp. nov.** is readily distinguished by its unlobed postpetiolar dorsum. Furthermore, new country records are presented: *C. acaciae* Forel for the KSA and Yemen, and *C. delagoensis* Forel and *C. jehovae* Forel for the KSA*C. antaris* for Qatar, whereas *C. luctans* Forel is excluded from the Arabian fauna. In addition, on the basis of morphological examination of original type material, *C. affabilis* Forel is proposed as junior synonym of *C. chiarinii* Mayr, and *C. striaticeps* is elevated to species rank **stat. nov.** Furthermore, a new identification key for the Arabian species is provided, as well as distribution maps for all species.

## Introduction

The myrmicine ant genus *Crematogaster* Lund, 1831 is one of the most species-rich genera of the family Formicidae with 500 described species, 269 valid subspecies and two fossil species (Blaimer 2012a; Bolton 2019). The genus is widely distributed worldwide throughout most of the tropical and subtropical regions (Hölldobler and Wilson 1990; Longino 2003). Most species are arboreal and build nests in dead branches, under bark or in carton nest structures, or, less commonly, nest directly in soil (Hölldobler and Wilson 1990; Madden and Young 1992; Palmer and Brody 2013). When natural history information is available, these ants are generalized foragers or omnivores with numerous species also tending homopterans and Hemipterans (e.g., Brown 2000; Campbell 1994; Gullan et al. 2018; Longino 2003) for honey dew.

Despite the remarkable diversity, ecological importance, and often high local abundance of the genus, it is one of the most taxonomically neglected hyper-diverse ant genera. Presently, the taxonomic situation is only moderate to satisfactory for a few regions, such as North America (Buren 1959; 1968), Costa Rica (Longino 2003), Madagascar (e.g., Blaimer 2010; 2012b; c), and parts of South East Asia (e.g., Hosoishi and Ogata 2008; 2009; 2015; 2017). However, apart from these, for most other regions, the taxonomy is in a very poor state, without modern revisionary treatments that cleared the taxonomic chaos from the 19^th^ and early 20^th^ century. As a consequence, the taxonomic validity of the majority of species and infraspecific taxa has not been comprehensively examined. We are certain that many nominally valid species and subspecies will have to be synonymized while at the same time there are numerous new species awaiting formal description. Therefore, species of *Crematogaster* are notoriously difficult to identify to species level in most parts of the world and the genus is in dire need of any modern, thorough revisionary study.

There are numerous, scattered, records of *Crematogaster* on the Arabian Peninsula. Prior to this study, the total number of species was 15 (plus one subspecies), recorded from the Kingdom of Saudi Arabia (KSA) (Collingwood 1985; Collingwood and Agosti 1996), the United Arab Emirates (UAE) (Collingwood and Agosti 1996; Collingwood et al. 2011), the Sultanate of Oman (Collingwood 1985; 1988; Collingwood and Agosti 1996; Sharaf et al. 2018), Yemen (Collingwood and van Harten 1994; 2001; 2005), and Kuwait (Collingwood and Agosti 1996). However, there are no records known from Bahrain, nor the Socotra Archipelago.

The taxonomy of Arabian *Crematogaster* is even more challenging than in other parts of the world due to the geographic location of the Arabian Peninsula, which connects sub-Saharan Africa or the Afrotropical region with the Mediterranean/Middle East or Palaearctic region (Kreft and Jetz 2010). As a consequence, the Arabian Peninsula shares biogeographical affinities with both, the Afrotropical and Palaearctic regions, however, no modern revisions of *Crematogaster* exist for these regions around the Arabian Peninsula. While there are some treatments covering particular countries or smaller areas in Europe or the northern Mediterranean, these either just describe new taxa or provide identification resources without revising the genus (Collingwood 1978, Agosti and Collingwood 1987, Cagniant 2005, Seifert 2007, Karaman 2008). In addition, many *Crematogaster* species have large female reproductives and good dispersal abilities leading to often vast distribution ranges. Thus, to be certain about the identity of any Arabian *Crematogaster* material it would require examining and comparing hundreds of Afrotropical and Palaearctic type specimens from numerous natural history collections. This enormous task is unfortunately outside the scope of our study; however, we believe this study can present a first important step towards understanding the species distributions of *Crematogaster* on the Arabian Peninsula.

We present a synoptic list, species accounts for all species that include detailed taxonomic histories, as well as data and maps showing the currently known distribution ranges. In addition, we also present a new identification key to the Arabian species of *Crematogaster* and describe two new species: *C. jacindae* sp. nov. from Oman and *C. gryllsi* sp. nov. from the Asir Mountains, KSA. Furthermore, we examine and propose some status changes for Arabian taxa and discuss doubts about identifications and species records for some species.

## Materials and methods

### Materials

Species names in this work follow the online catalogue of Bolton (2019) and in addition to published references (e.g., Collingwood 1985; Collingwood and Agosti 1996; Hita Garcia et al. 2013). The taxonomic histories of the treated taxa follow the online catalogue of ants of the world (Bolton 2019) and Antwiki (www.antwiki.org). Digital stacked color images were created using a Leica DFC450 digital camera with a Leica Z16 APO microscope and LAS (v3.8) software. These images are also available online on AntWeb (https://www.antweb.org) and are accessible through unique specimen identifiers attached (e.g., CASENT0872096). Distribution ranges were examined on Antmaps (https://antmaps.org; Guénard et al. 2017; Janicki et al. 2017) and general information of species on Antwiki. Distribution maps were made using DIVA-GIS (version 7.5.0.0).

### Terminology and measurements

The terminology used to describe surface sculpture is based on (Harris 1979). Morphological terminology, measurements, and indices follow (Blaimer 2010, Longino 2003).

**EL** Eye length; maximum diameter of compound eye in profile.

**HL** Head length; maximum distance from the midpoint of anterior clypeal margin to midpoint of posterior margin of head, measured in full-face view.

**HW** Head width: maximum width of head behind eyes in full-face view.

**LHT** Length of metatibia, excluding the proximomedial condyle.

**ML** Mesosomal length; diagonal length of mesosoma in profile from posteroventral margin of propodeal lobe to anterior most point of pronotal slope, excluding neck.

**PTH** Petiole height; measured from petiole sternum to apex in profile.

**PPL** Postpetiole length: maximum length of postpetiole measured in dorsal view.

**PPW** Postpetiole width: maximum width of postpetiole measured in dorsal view.

**PRW** Pronotal width: maximum pronotal width in dorsal view.

**PTL** Petiole length: measured in profile as distance from dorso-posterior margin of segment to anterior inflection point where petiole curves up to condyle.

**PTW** Petiole width: maximum width of dorsal face of petiole node measured in dorsal view.

**SL** Scape length; maximum scape length excluding basal condyle and neck.

### Indices

**OI** Ocular index: EL / HW × 100

**CI** Cephalic index: HW / HL × 100

**SI** Scape index: SL / HW × 100

**PTHI** Petiole height index: PTH / PTL × 100

**PTWI** Petiole width index: PTW / PTL × 100

**LBI** Leg-body index: WL / LHT × 100

**PPI** Postpetiole index: PPW / PTW × 100

Throughout the text, ‘**w**’ stands for ‘worker’ or ‘workers’, ‘**q**’ for queen, ‘**m**’ for male, ‘**BS**’ for beating sheet, ‘**ML**’ for Malaise trap, and ‘**SF**’ sifting.

### Institutions and museums

The abbreviations of natural history collections follow Evenhuis (2019) and Brandão (2000).

**BMNH** The Natural History Museum, London, U.K.

**CASC**California Academy of Sciences collection, California Academy of Sciences, San Francisco, California, USA.

**KSMA**King Saud University Museum of Arthropods, Plant Protection Department, College of Food and Agriculture Sciences, King Saud University, Riyadh, Kingdom of Saudi Arabia.

**MHNG**Muséum d’Histoire Naturelle, Geneva, Switzerland.

**OUMC** Oxford University Museum, Oxford, U.K.

**WMLC** World Museum Liverpool, Liverpool, U.K.

## Results

### Synoptic list of Arabian species of *Crematogaster*

*Crematogaster acaciae* Forel, 1892

*Crematogaster aegyptiaca* Mayr, 1862

*Crematogaster antaris* Forel, 1894b

*Crematogaster auberti* Emery, 1869a

*Crematogaster chiarinii* Emery, 1881

= *Crematogaster affabilis* Forel, 1907b syn. nov.

*Crematogaster delagoensis* Forel, 1894a

*Crematogaster flaviventris* Santschi, 1910

*Crematogaster gryllsi* Sharaf & Hita Garcia sp. nov.

*Crematogaster inermis* Mayr, 1862

*Crematogaster jacindae* Sharaf & Hita Garcia sp. nov.

*Crematogaster jehovae* Forel, 1907c

*Crematogaster laestrygon* Emery, 1869b

*Crematogaster striaticeps* Forel, 1909, stat. nov.

*Crematogaster melanogaster* Emery, 1895

*Crematogaster mimosae* Santschi, 1914a

*Crematogaster oasium* Santschi, 1911

*Crematogaster senegalensis* Roger, 1863

### Identification key to Arabian species of *Crematogaster*

**Table:** 

1	Postpetiole not bilobed dorsally (Figs 1A, 17A–C)	***C. gryllsi* sp. nov.**
–	Postpetiole bilobed dorsally (Fig. 1B)	**2**
2	Propodeal spines completely absent (Fig. 1C)	**3**
–	Propodeal spines present, ranging from small denticles to long spines (Fig. 1D)	**4**
3	Unicolorous yellow-brown to brown species; cephalic surface including area in front of eyes unsculptured; eyes with ca. 14 ommatidia in longest row; posterior half of clypeus with fine appressed pubescence; mesonotum in profile with a small tubercle close to promesonotal suture (Fig. 1E); mesopleura and metapleura longitudinally striated (Fig. 1E); mesonotum without hairs; propodeal spiracles circular (Figs 1E, 20A–C)	***C. inermis* Mayr**
–	Bicolored species, head black-brown or black, mesosoma, petiole and postpetiole dark brown, relatively lighter than head, gaster golden yellow; area in front of eyes finely longitudinally striated; cephalic surface feebly imbricate; eyes with ca. 11 ommatidia in longest row; posterior half of clypeus without hairs or pubescence; mesonotum in profile without tubercle (Fig. 1F); mesopleura and metapleura distinctly densely imbricate (Fig. 1F); mesonotum with a single pair of hairs; propodeal spiracles slit-shaped (Fig. 1F), gaster golden yellow (Fig. 21A–C)	***C. jacindae* sp. nov.**
4	Propodeal spines reduced to a small denticle (Fig. 14A–C)	***C. delagoensis* Forel**
–	Propodeal spines well developed	**5**
5	In full-face view, antennal scapes short, clearly not reaching posterior margin of head (Fig. 2A)	**6**
–	In full-face view, antennal scapes longer, clearly reaching or surpassing posterior margin of head (Fig. 2B)	**9**
6	Propodeal spines short and blunt (Fig. 2C), approx. as long as their bases in profile; antennal fossae surrounded by curved striolae in full-face view (Figs 2A, 7A–C)	***C. aegyptiaca* Mayr**
–	Propodeal spines long and acute, distinctly longer than their bases in profile (Fig. 2D); antennal fossae surrounded by longitudinal striolae in full-face view (Fig. 2E)	**7**
7	Body distinctly opaque; cephalic surface completely densely longitudinally rugulose (Fig. 2F); head, mesosoma, petiole and postpetiole reddish (Fig. 27A–C)	***C. mimosae* Santschi**
–	Body shining; only area in front of eyes faintly longitudinally rugolose; head, mesosoma, petiole and postpetiole uniform yellow or brown	**8**
8	Body uniform yellow; head, in full-face view, with feebly-defined frontal triangle and without longitudinal carina; postpetiole in dorsal view broader posteriorly than anteriorly; propodeum dorsum seen from above longitudinally striated (Fig. 5A–C)	***C. acaciae* Forel**
–	Head, petiole, postpetiole and gaster dark brown, mesosoma light brown; head, in full-face view, with well-defined frontal triangle and posterior longitudinal carina reaching posterior margin of eyes; propodeum dorsum seen from above transversally striated; postpetiole in dorsal view as broad as anteriorly and posteriorly (Fig. 12A–C)	***C. chiarinii* Emery**
9	Antennal scapes when laid back from their insertions just reach posterior margin of head in full-face view	**10**
–	Antennal scapes when laid back from their insertions clearly surpassing posterior margin of head in full-face view	**11**
10	Unicolorous brown species; clypeus smooth; anterior half of head in full-face view longitudinally striated, ground surface between striae smooth (Fig. 3A); posterior half of head smooth, general appearance of head shining (Fig. 29A–C)	***C. senegalensis* Roger**
–	Bicolored species, head, mesosoma, petiole, postpetiole and appendages brown or red-brown, gaster golden yellow; clypeus longitudinally striated; anterior half of head in full-face view finely longitudinally striated, grown surface between striae and posterior half of head finely densely punctate (Fig. 3B); general appearance of head dull (Fig. 15A–C)	***C. flaviventris* Santschi**
11	Mesonotum in profile without a small tubercle close to promesonotal suture	**12**
–	Mesonotum in profile with a small tubercle close to promesonotal suture	**13**
12	Whole cephalic surface finely, densely longitudinally striated and dull; head in full-face view with antennal scapes surpassing posterior margin of head byapproximate length of the three funicular segments together (Fig. 3C); propodeal spines long and acute, more than twice longer than bases (Fig. 3D); mesonotum in profile feebly convex (Fig. 3D); subpetiolar process well developed (Fig. 3D); mesosomal hairs numerous (Fig. 3D), five on promesonotum, two on pronotum, and a single pair on the propodeum (Fig. 26A–C)	***C. melanogaster* Emery**
–	Anterior half of cephalic surface superficially striated, posterior half smooth and shining; head in full-face view with antennal scapes surpassing posterior margin of head by ca. thickness of the first funicular segment (Fig. 3E); propodeal spines shorter and blunt, ca. 1.5× longer than bases (Fig. 3F); mesonotum in profile strongly convex (Fig. 3F); subpetiolar process absent; mesosomal hairs restricted to a single pair on pronotum (Fig. 10A–C)	***C. auberti* Emery**
13	Cephalic surface and clypeus completely finely densely longitudinally striated (Fig. 30A–C)	***C. striaticeps* Forel**
–	Anterior half of cephalic surface or at least area in front of eyes and clypeus longitudinally striated	**14**
14	Promesonotum with at least four pairs of suberect hairs; petiole in dorsal view with pointed anterior corners; first gastral tergite with several pairs of hairs (ca. 7 pairs) (Fig. 28A–C)	***C. oasium* Santschi**
–	Promesonotum with a single pair of hairs or without hairs; petiole in dorsal view with rounded anterior corners; hairs on first gastral tergites rare, restricted to few pairs on posterior margin of the tergite	**15**
15	Petiole in dorsal view with concave anterior margin (Fig. 4A); promesonotum and mesonotum dorsum unsculptured (Fig. 8A–C)	***C. antaris* Forel**
–	Petiole in dorsal view with a straight anterior margin (Fig. 4B); promesonotum and mesonotum dorsum distinctly longitudinally striated	**16**
16	Postpetiole approx. twice broader than long in dorsal view (Fig. 4C); color black-brown or dark brown (Fig. 24A–C)	***C. laestrygon* Emery**
–	Postpetiole little broader than long in dorsal view (Fig. 4D); color red-brown (Fig. 23A–C)	***C. jehovae* Forel**

**Figure 1.**
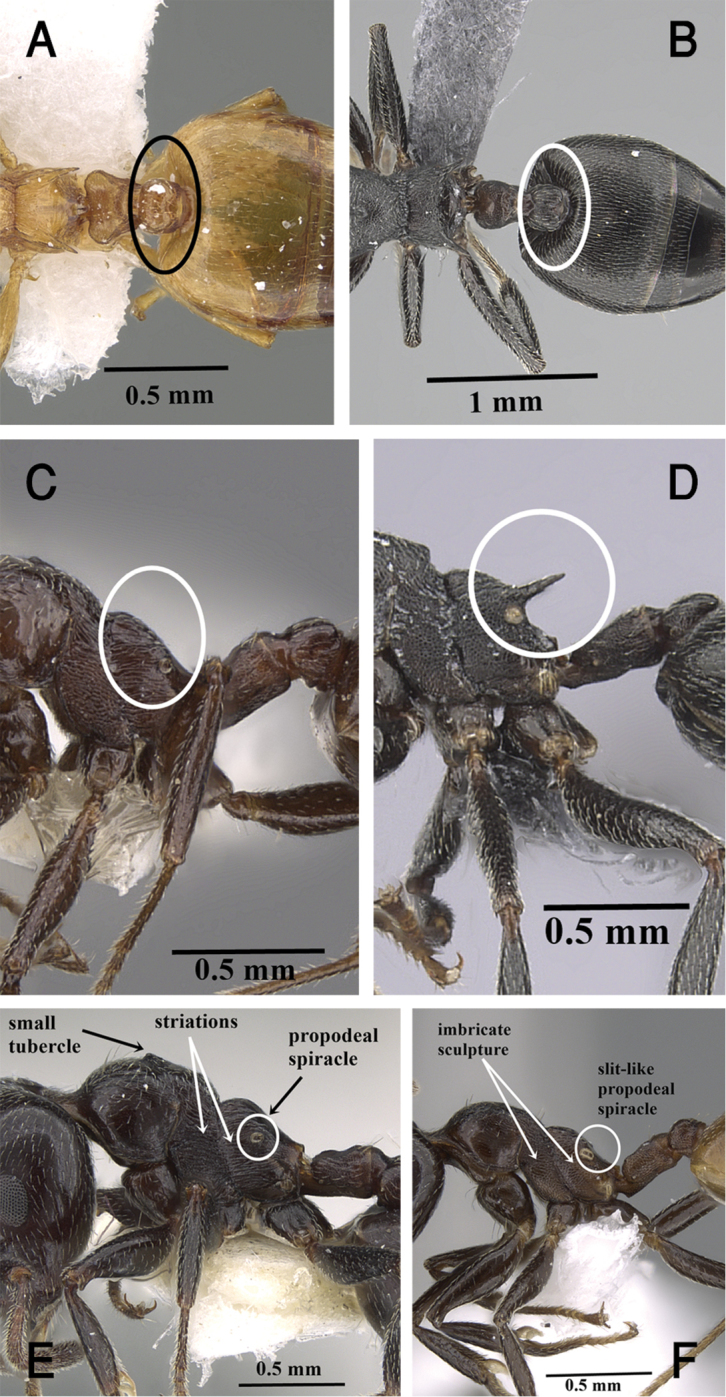
**A** Postpetiole of *C. gryllsi* sp. nov. in dorsal view, CASENT0919794 (Michele Esposito) **B** postpetiole of *C. chiarinii* in dorsal view, CASENT0263878 (Will Ericson) **C** propodeum of *C. inermis* in profile, CASENT0922679 (Wade Lee) **D** propodeal spines of *C. chiarinii* in profile, CASENT0263878 (Will Ericson) **E** mesosoma of *C. inermis* in profile, KG01956A (Michele Esposito) **F** mesosoma of *C. jacindae* in profile CASENT0922856 (Michele Esposito), www.AntWeb.org.

**Figure 2.**
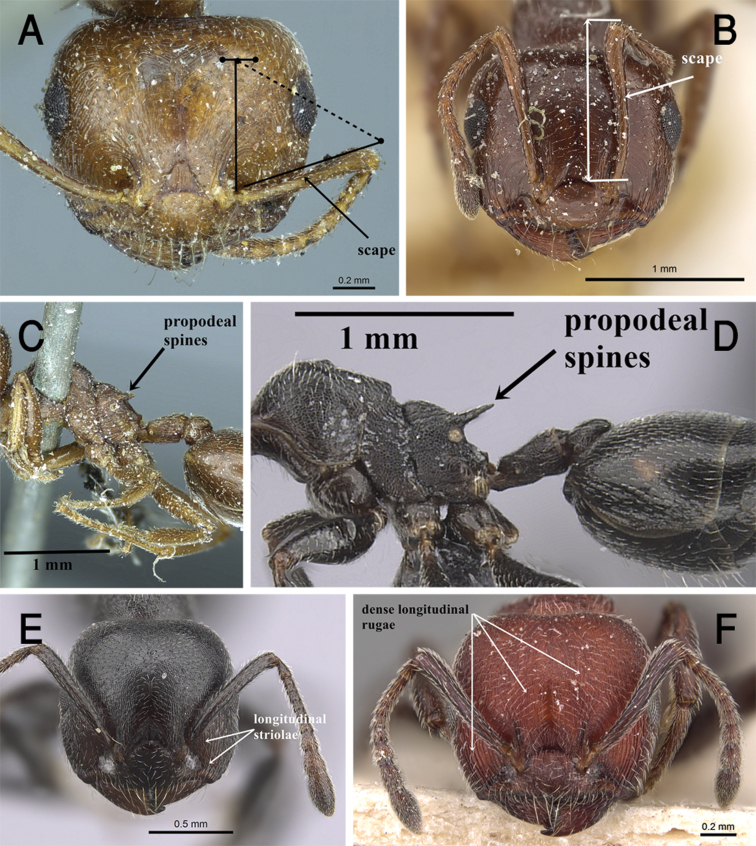
**A** Head of *C. aegyptiaca* in full-face view, CASENT0916082 (Anna Pal) **B** head of *C. oasium* in full-face view, CASENT0249821 (Ryan Perry) **C** mesosoma of *C. aegyptiaca* in profile, CASENT0916082 (Anna Pal) **D** propodeal spines of *C. chiarinii* in profile, CASENT0263878 (Will Ericson) **E** head of *C. chiarinii* in full-face view, CASENT0263878 (Will Ericson) **F** head of *C. mimosae* in full-face view,CASENT0904507 (Will Ericson), www.AntWeb.org.

**Figure 3.**
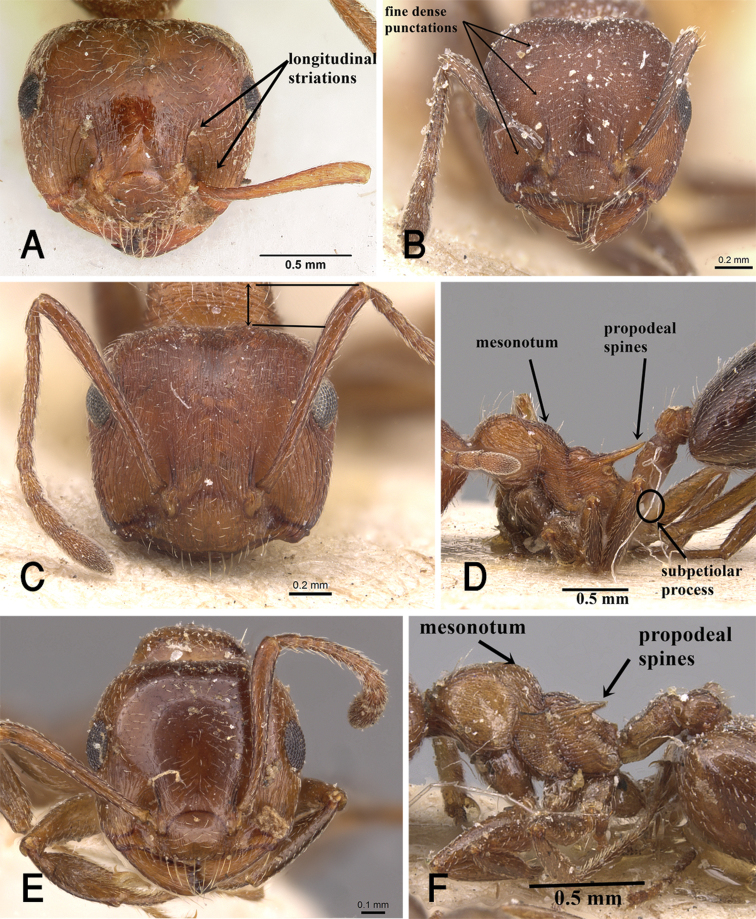
**A** Head of *C. senegalensis* in full-face view, CASENT0104592 (April Nobile) **B** head of *C. flaviventris* in full-face view, CASENT0912651 (Will Ericson) **C** head of *C. melanogaster* in full-face view, CASENT0904511 (Will Ericson) **D** mesosoma of *C. melanogaster* in profile, CASENT0904511 (Will Ericson) **E** head of *C. auberti* in full-face view, CASENT0908480 (Zach Lieberman) **F** mesosoma of *C. auberti* in profile, CASENT0904499 (Will Ericson), www.AntWeb.org.

**Figure 4.**
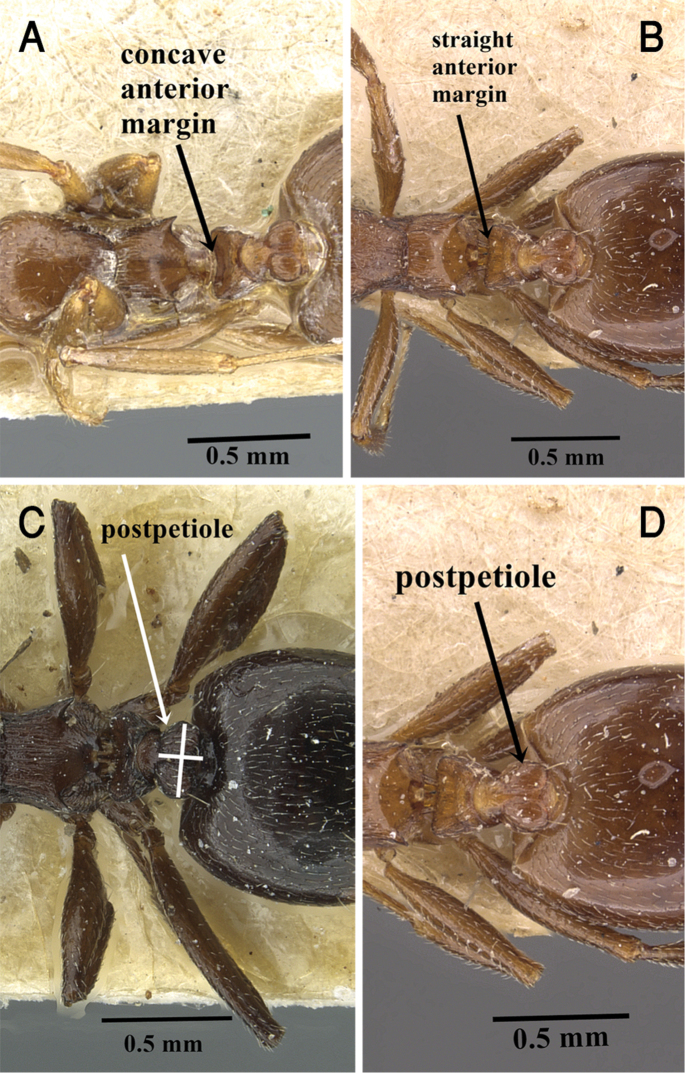
**A** Petiole of *C. antaris* in dorsal view, CASENT0903658 (Will Ericson) **B** petiole of *C. jehovae* in dorsal view, CASENT0908475 (Zach Lieberman) **C** postpetiole of *C. laestrygon* in dorsal view, CASENT0912691 (Zach Lieberman) **D** postpetiole of *C. jehovae* in dorsal view, CASENT0908475 (Zach Lieberman), www.AntWeb.org.

### Review of Arabian species

#### Crematogaster acaciae

Taxon classificationAnimaliaHymenopteraFormicidae

Forel

77C5AECA-756D-5B9F-BCF6-2EDD62BE9934

Figure 5A–C

##### Taxonomic history.

*Crematogaster acaciae* Forel, 1892: 141 (w.) Ethiopia.

Combination in Crematogaster (Acrocoelia): Emery 1922: 144; in *Crematogaster*. (*Crematogaster*): Bolton 1995: 166.

Subspecies of *C. brunneipennis*: Wheeler 1922: 152.

Status as species: Emery 1922: 144; Collingwood 1985: 260.

Current subspecies: *C. acaciae generosa* Santschi, *C. acaciae gloriosa* Santschi, *C. acaciae victoriosa* Santschi.

##### Material examined.

**KSA**: Jebel Dhablah, 27.79175N, 41.34063E, 03.v.1985, 950 m (W. Buttiker) (1 w, WMLC); Alqatif, 26.51028N, 49.96889E, 14.iv.1983 (Collingwood CA) (1 w, WMLC); Alqatif, 26.51028N, 49.96889E, 15.iv.1983 (Collingwood CA) (48 w, WMLC); **Oman**: muqshen (mugshin), 19.55N, 54.883333E, 20.ix.1979 (R. W. Whitcomb) (1 w, WMLC); 67 km S. Mintirib, desert trade, 27.i.1986 (W. Buttiker) (9 w, eastern sand project, WMLC); W. of Muscat, 23.588N, 58.3829E, 26.ix.1982 (M. Gallagher) (2w, 6374, WMLC); **Yemen**: Al Kawd, 13.088622N, 45.364722E, x.1992 (1 q, WMLC); **Namibia**: Kuzikus Wildlife Reserve, Windhoek, 23.2306S, 18.401617E, 1340 m, 07.ix.2009 (H Campbell) (I W, OUMC); **Sudan**: Khordonia, 11.85N, 34.25E, 24.x.2001 (J. Mathews) (2 W, OUMC); Khordonia, Damazine Plantation, Blue Nile, 11.85N, 34.25E, 25.x.2000 (J. Mathews) (2 W, OUMC); **Tanzania**: Mkomazi Igire Hill, 10.i.1996 (G. McGavin) (2 W, OUMC).

##### Geographic range.

*Crematogaster acaciae* was originally described from Ethiopia but is also known from Democratic Republic of Congo, Somalia, South Africa, and Zambia (Guénard et al. 2017; Janicki et al. 2017). For the Arabian Peninsula, it was previously only known from Oman (Collingwood 1985; Collingwood and Agosti 1996; Borowiec 2014; Sharaf et al. 2018; Monks et al. 2019) (Fig. 6). In this study, we provide the first records for the KSA and Yemen.

##### Remarks.

This species represents a typical problematic taxon within the genus. The known distribution of *C. acaciae* and its three subspecies in the Afrotropics is patchy and there are some notable morphological differences between the infraspecific taxa. At present, it is likely that the taxonomic status of the involved taxa will change within the frame of a comprehensive revision of the Afrotropical fauna.

**Figure 5.**
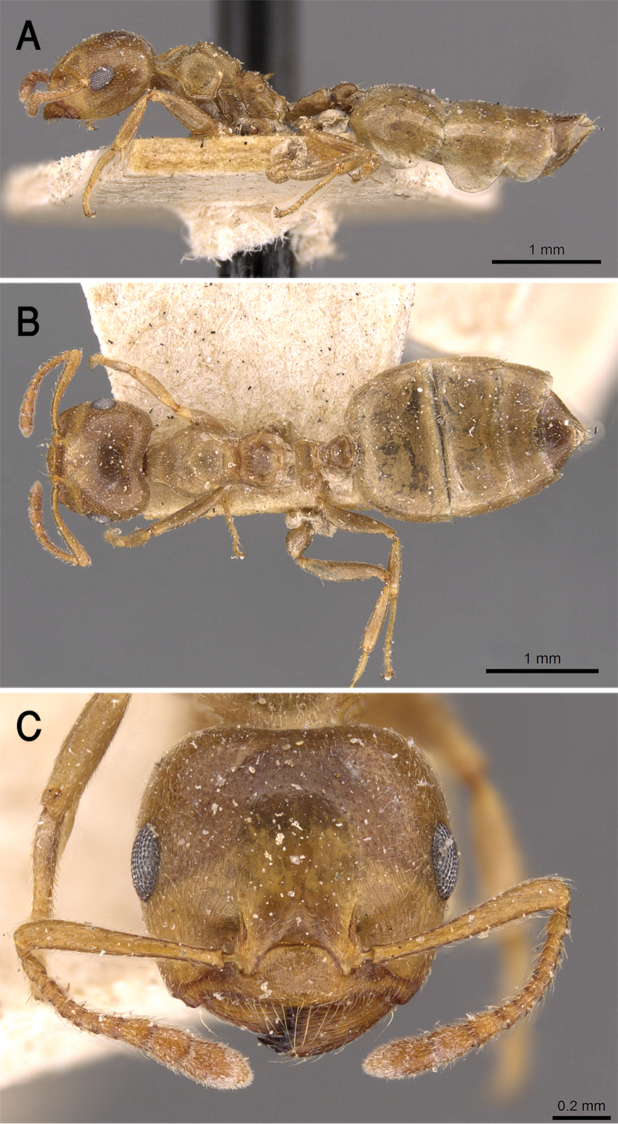
*C. acaciae***A** body in profile **B** body in dorsal view **C** head in full-face view, CASENT0908494 (Zach Lieberman), www.AntWeb.org.

**Figure 6.**
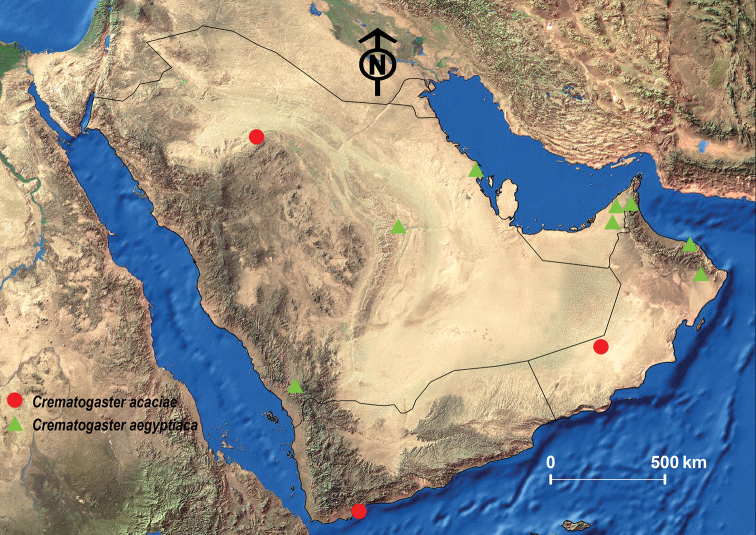
Distribution map of *C. acacia* and *C. aegyptiaca*.

#### Crematogaster aegyptiaca

Taxon classificationAnimaliaHymenopteraFormicidae

Mayr

FA625A9E-D334-5422-8256-893CC7069846

Figure 7A–C

##### Taxonomic history.

*Crematogaster aegyptiaca* Mayr, 1862: 765 (w.) Egypt.

Combination in Crematogaster (Crematogaster): Wheeler 1922: 828; in Crematogaster (Acrocoelia): Emery 1922: 144; in Crematogaster (Crematogaster): Bolton 1995: 166.

Current subspecies: *C. aegyptiaca pharaonis* Santschi, *C. aegyptiaca robusta* Emery, *C. aegyptiaca turkanensis* Santschi.

##### Material examined.

**KSA**: Asir Province, Abha, Al Habala, 18.034167N, 42.858167E, 2397 m, 25.iv.2011 (Sharaf MR) (19 w, KSMA); Riyadh, Al Hayer, 24.290833N, 46.9075E, 10.iii.2011 (Sharaf MR) (14 w, KSMA); **UAE**: Near Mahafiz, 25.09N, 55.48E, 24.iii-07.iv.2011 (M Hauser et al.), UAE13055 (1 w, CASENT0264387, 1 w, CASENT0264388, KSMA); Wadi Maidaq, 25.18N, 56.07E, 15–31.x.2010 (M Hauser et al.), UAE12848 (1 w, CASENT0265207, KSMA); **Oman**: dunes near Mintirib, 22.4248N, 58.8032E, 17.xi.1984 (M. Gallagher) (1 w, WMLC).

##### Geographic range.

This species was originally described from Egypt and can be found in most most countries of North Africa but also in Sudan and Kenya (Guénard et al. 2017; Janicki et al. 2017). For the Arabian Peninsula, it was recorded from the KSA, Oman, the UAE, and Yemen (Borowiec 2014; Collingwood 1985, Collingwood and Agosti 1996; Collingwood et al. 2011; Sharaf et al. 2018) (Fig. 6).

##### Remarks.

As for *C. acacia* (and many other taxa), the taxonomic condition of this species is relatively unclear due to the absence of any revisions in the regions in question, and we cannot predict the taxonomic status of *C. aegyptiaca* and its subspecies after being revised. However, based on our experience with this species, it is readily identifiable and we are very confident of the Arabian material being genuine *C. aegyptiaca*.

**Figure 7.**
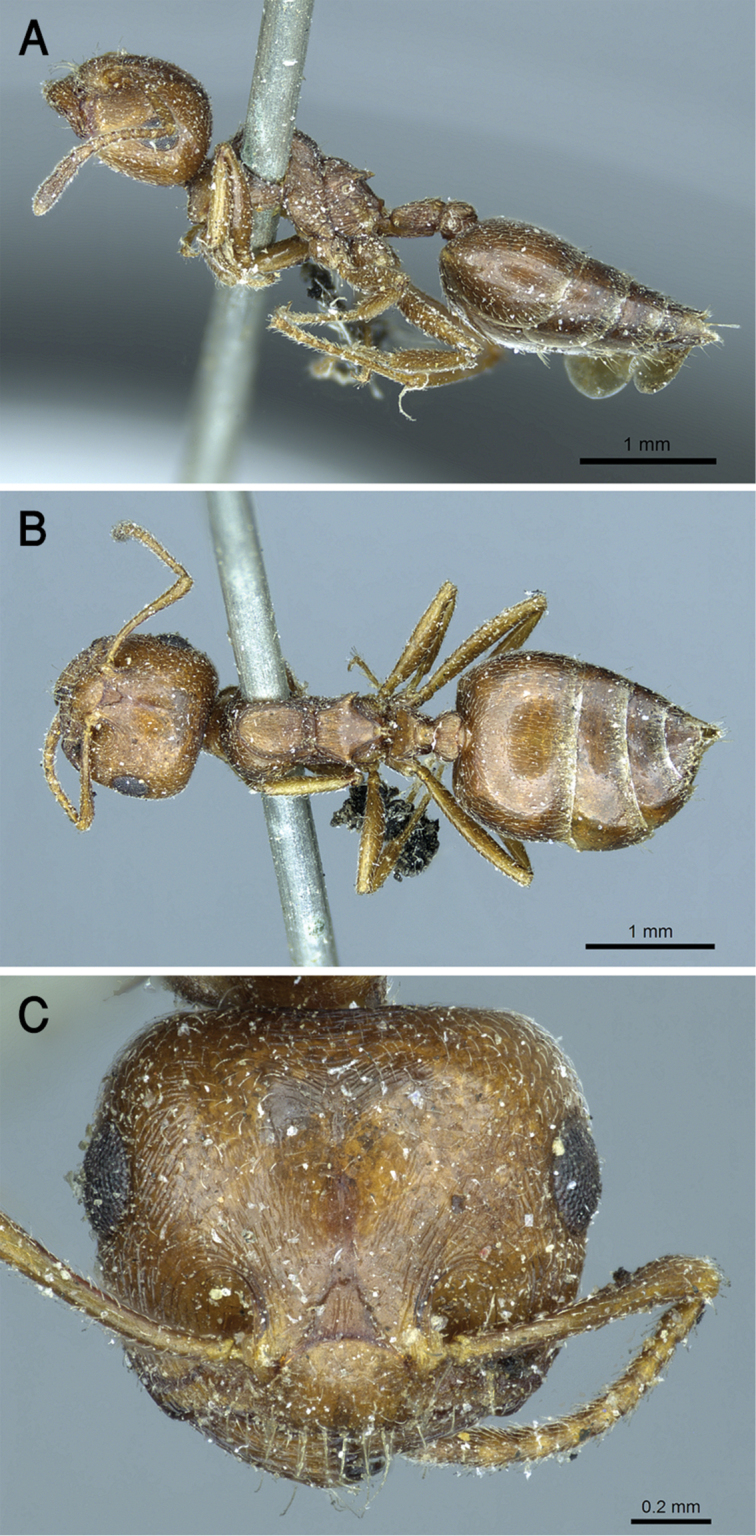
*C. aegyptiaca***A** body in profile **B** body in dorsal view **C** head in full-face view, CASENT0916082 (Anna Pal), www.AntWeb.org.

#### Crematogaster antaris

Taxon classificationAnimaliaHymenopteraFormicidae

Forel

20540D5E-0831-5066-8A7E-0DA863FE3DA4

Figure 8A–C

##### Taxonomic history.

Crematogaster (Acrocoelia) auberti r. antaris Forel, 1894b: 26 (w., q.) Algeria.

Combination in Crematogaster (Acrocoelia): Emery 1922: 142; in Crematogaster (Crematogaster): Bolton 1995: 166.

Subspecies of *C. inermis*: Emery 1926: 2; Menozzi 1927: 379; of *C. auberti*: Emery 1924: 8; Finzi 1930: 15; Santschi 1938: 38; Cagniant 1964: 103.

Status as species: Santschi 1921: 71; Collingwood 1985: 260.

Crematogaster auberti var. sordida Forel, 1909: 104 (w.) Algeria. [First available use of Crematogaster auberti r. laestrygon var. sordida Forel, 1894: 26; unavailable name]. Combination in C. (Acrocoelia): Emery 1922: 142.

Subspecies of *C. antaris*: Santschi 1921: 71. Junior synonym of *C. antaris*: Cagniant 2005: 11.

##### Material examined.

**KSA**: Riyadh, Al Mezahmyiah, 24.46633N, 46.25131E, 648 m, 29.xi.2014 (Salman S) (19 w, KSMA); Riyadh, Al Dawadmy, 24.55216N, 43.93170E, 873 m, 16.i.2015 (Salman S) (9 w, KSMA); Taif, Al Wesam district, 21.204722N, 40.345278E, 11.x.2010 (Al Dhafer et al.) (5 w, KSMA); Asir province, Ballasmer, A’l Azza, 18.60815N, 42.24628E, 2611 m, 27.iv.2019 (Sharaf MR) (2 w, KSMA); Asir province, Abha, Al Souda, 18.274167N, 42.364444E, 2982 m, 24.iv.2011 (Sharaf MR) (1 w, KSMA); Taif, Shafa of Shafa hwy, 21.139167N, 40.351389E, 12.x.2010 (Al Dhafer et al.) (8 w, KSMA); Altawil, 18.016667N, 42.95E, 21–22.ix.1984 (W. Buttiker) (1 w, WMLC); W. Harith, 17.4863N, 44.0825E, 28.ix.1978 (W. Buttiker) (1 w, WMLC); El Shoiba, 20.680084N, 39.523233E, 28.xii.1978 (W. Buttiker) (1 w, WMLC); Jeddah, 21.4858N, 39.1925E, 26.ii.1934 (G. L. Bates, B.M. 1934-404) (6 w, BMNH). **Oman**: Mintrib, 22.4248N, 58.8032E (M. D. Gallagher) (7 w, WMLC); Qarhat Mu’ammur, 21.666667N, 59.316667E, 22.iv.1986 (M. D. Gallagher) (1 w, 7691, WMLC); khabura (Al Khaburah), 23.9628N, 57.0957E, 10.iii.1980 (R. W. Whitcomb) (1 w, WMLC); Musandan, 26.03333N, 56.3E (3 w, WMLC); Mu’ammur, 1986 (W. Buttiker) (1 q, WMLC); Um Qashab, 27.iv.1986 (M. D. Gallagher) (2 w,7865, WMLC); 50–90 km E of Hayam, ii-iii.1993 (R.D. Schumann) (1 w, desert, in spirit tube, WMLC); Qarhat Mu’ammur, 21.666667N, 59.316667E, 135 m, 02.ii.1986 (W. Buttiker) (4 w in spirit tube, eastern sand project, dunes, WMLC); Khabura, 23.9628N, 57.0957E, 01.xii.1979 (R. W. Whitcomb) (6 w, BMNH); Rustaq, 23.387831782N, 57.421331648E, 07.v.1979 (R. W. Whitcomb) (11 w, BMNH); Khboura, 23.9628N, 57.0957E, 27.i.1980 (R. W. Whitcomb) (21 w, BMNH). **Qatar**: Umsaid Road, 09.iv.2005 (M. S. Abdel-Dayem) (10 w, KSMA); Al Kharara-Seleiyn Road, 09.iv.2005 (M. S. Abdel-Dayem) (13 w, KSMA). **UAE**: Ras Ghanada, vi.1995 (B. Tigar) (15 w in spirit tube, WMLC); Public Hunting Ground, 12.viii.1994 (B. Tigar) (13 w in spirit tube, 2360, WMLC); Al Ain Zoo, 24.1792N, 55.7396E, iv-v.1995 (B. Tigar) (7 w in spirit tube, WMLC); Sweihan, 24.4582N, 55.3324E, 04.iii.1995 (Collingwood CA) (25 w in spirit tube, stony desert, WMLC); Khour Al Taafirah, 17.2N, 42.35E, 10.x.1984 (W. Buttiker) (14 w in spirit tube, WMLC); Dubai city, 25.2048N, 55.2708E, 2002 (K. Valsan) (4 w, BMNH). **Yemen**: W. Aden Port, Jebel Jihaf, 04.x.1937 (H. Scott & E. B.Britton. B.M.1933-246) (9 w, B. M. Expedition to S.W. Arabia, BMNH); Summit of Jebel Kohl,15 miles N. of Sana’a, 01.ii.1938 (H. Scott & E. B. Britton. B.M.1933-246) (6 w, B.M. Expedition to S.W. Arabia, H. Scott & E. B. Britton, B.M.1933-246).

##### Geographic range.

Originally described from Algeria, *C. antaris* is also found in Morroco, Tunisia, Egypt, and Iran (Sharaf 2006; Paknia et al. 2008; Vonshak and Ionescu-Hirsch 2009; Borowiec 2014; Guénard et al. 2017; Janicki et al. 2017). Its apparent absence in Libya is likely a sampling artifact. *Crematogaster antaris* is also broadly distributed in the Arabian Peninsula where it is known from Kuwait, Oman, the KSA, the UAE, and Yemen (Collingwood 1985; Tigar and Collingwood 1993; Collingwood and Agosti 1996; Collingwood et al. 2011; Borowiec 2014; Sharaf et al. 2018) (Fig. 9).

##### Remarks.

This is a very widespread and common species, which appears to be one of the most arid-adapted species within the genus. Our collections represent a new record for Qatar.

**Figure 8.**
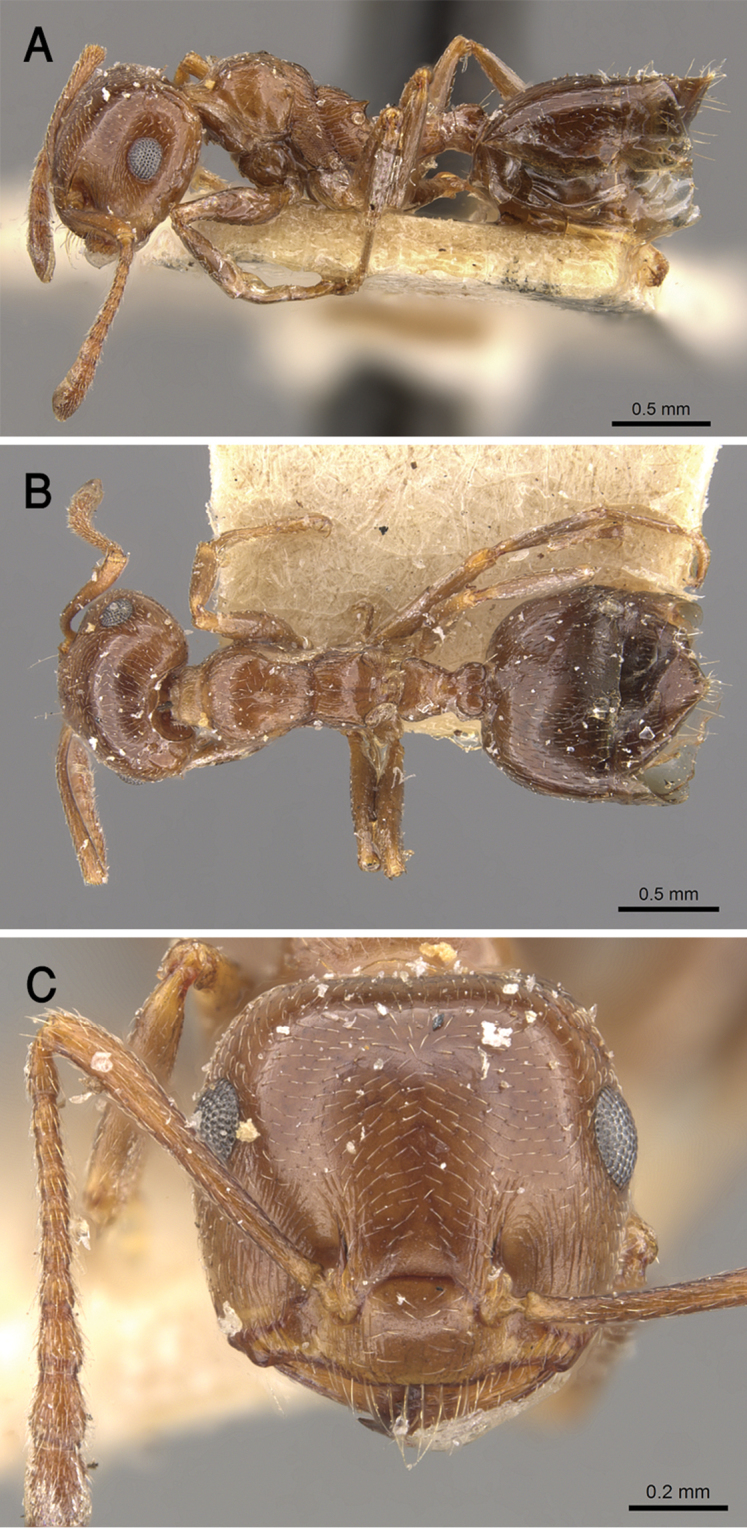
*C. antaris***A** body in profile **B** body in dorsal view **C** head in full-face view, CASENT0908473 (Will Ericson), www.AntWeb.org.

**Figure 9.**
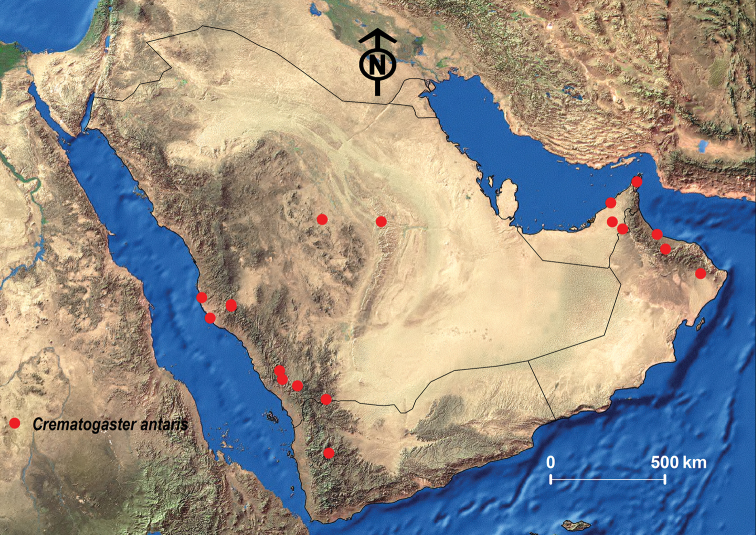
Distribution map of *C. antaris*.

#### Crematogaster auberti

Taxon classificationAnimaliaHymenopteraFormicidae

Emery

2D5F2813-AFDD-5F15-8538-D62452A6CE8F

Figure 10A–C

##### Taxonomic history.

Crematogaster (Acrocoelia) auberti Emery, 1869a: 23 (footnote) (w.) FRANCE; André 1883: 395 (q.); Emery 1891: 14 (m.).

Combination in Crematogaster (Acrocoelia): Emery 1922: 142; in Crematogaster (Crematogaster): Bolton 1995: 166.

Subspecies of *C. scutellaris*: Emery and Forel 1879: 464; of *C. schmidti*: Emery 1891: 14; of *C. inermis*: Emery 1926: 3.

Status as species: Forel 1894b: 24; Forel 1902: 152; Bondroit 1918: 115; Karavaiev 1927: 104; Finzi 1930: 15; Santschi 1937: 300.

Current subspecies: *C. auberti karawaewi* Ruzsky, *C. auberti levithorax* Forel, *C. auberti nigripes* Menozzi, *C. auberti regilla* Santschi, *C. auberti savinae* Zimmermann, *C. auberti vogti* Forel.

Senior synonym of Crematogaster auberti var. iberica Forel, 1909: 103 (w.) SPAIN.

Junior synonym of *C. auberti*: Collingwood 1978: 69.

##### Material examined.

**KSA**: Riyadh, Al Ammaryia, 24.794167N, 46.423333E, 648 m, 21.i.2010 (Aldawood AS) (12 w, KSMA); Eastern province, Buqaiq, 25.962222N, 49.647139E, 95 m, 03.iii.2011 (Al Dhafer et al.) (2 w, KSMA); **Egypt**: Alexandria, 07.v.1899 (1w, BMNH); **Algeria**: Bona, 24.xi.1993 (2 w, BMNH); **Morocco**: Tamri N. de Agadir, 13–17.iii.1961 (Meinander) (3 w, BMNH).

##### Geographic range.

This species was initially described from France and seems to have a broad distribution range from the south of France, the Iberian Peninsula, and the Canary Islands through all of North Africa to the Middle East, but is also found throughout the Balkans (Sharaf 2006; Borowiec and Salata 2012; Borowiec 2014; Guénard et al. 2017; Janicki et al. 2017). On the Arabian Peninsula it has been only recorded from the KSA (Collingwood 1985; Collingwood and Agosti 1996; Borowiec and Salata 2012; Borowiec 2014) (Fig. 11).

##### Remarks.

This is likely one of the most problematic species treated in this study. The taxonomic history provided above is complex with numerous status changes and infraspecific taxa. Borowiec (2014) states that all records from the eastern parts of the Mediterranean region are doubtful and likely misidentifications. If so, this would mean that the material from KSA is probably another species. However, at present and without a meaningful treatment of the European *Crematogaster* fauna, it is not possible for us to ascertain the genuine identity of this species. Thus, we maintain it as *C. auberti* for the moment and await further study of type material in European collections which might help elucidate its real identity.

**Figure 10.**
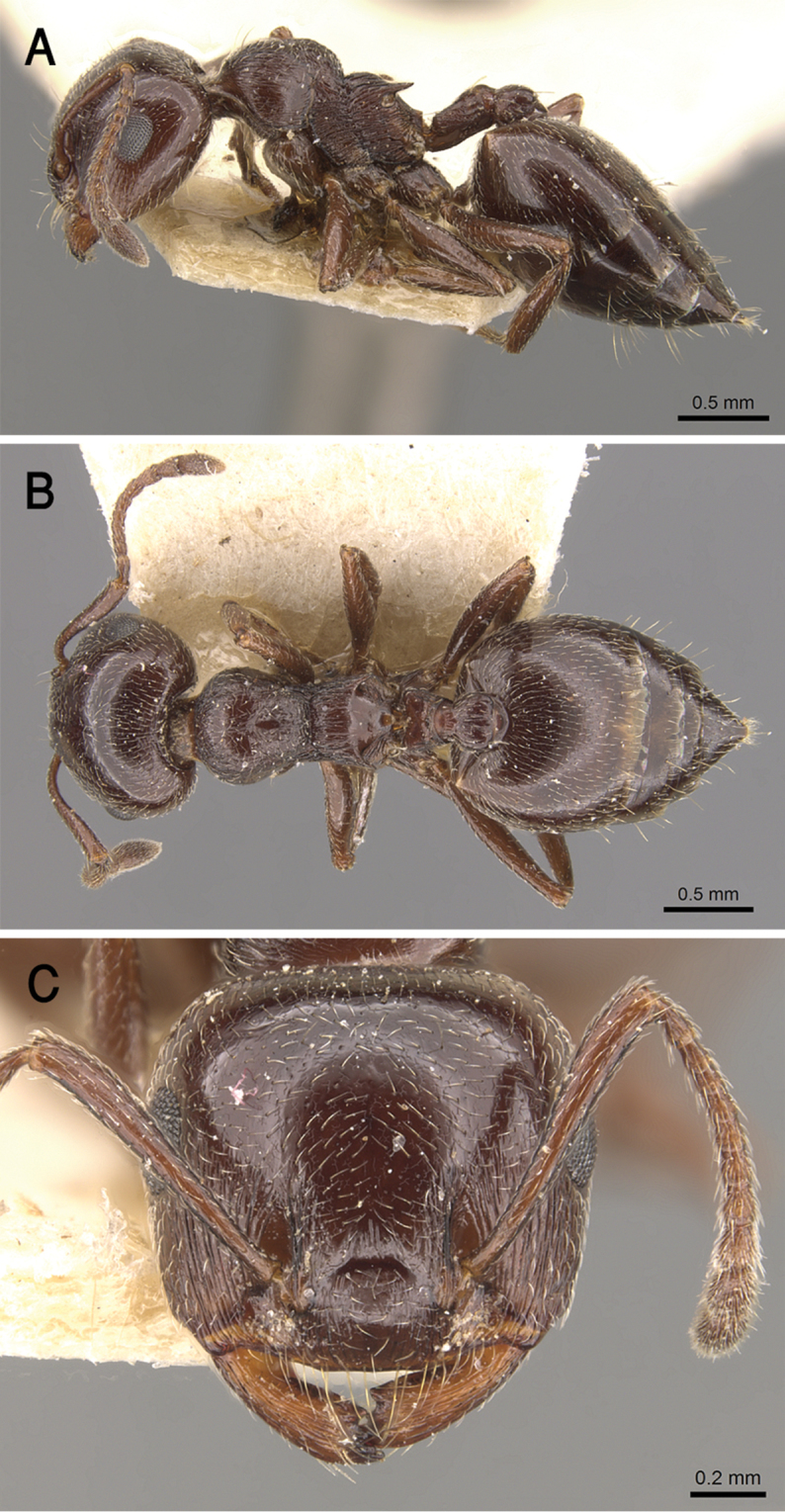
*C. auberti***A** body in profile **B** body in dorsal view **C** head in full-face view, CASENT0908470 (Will Ericson), www.AntWeb.org.

**Figure 11.**
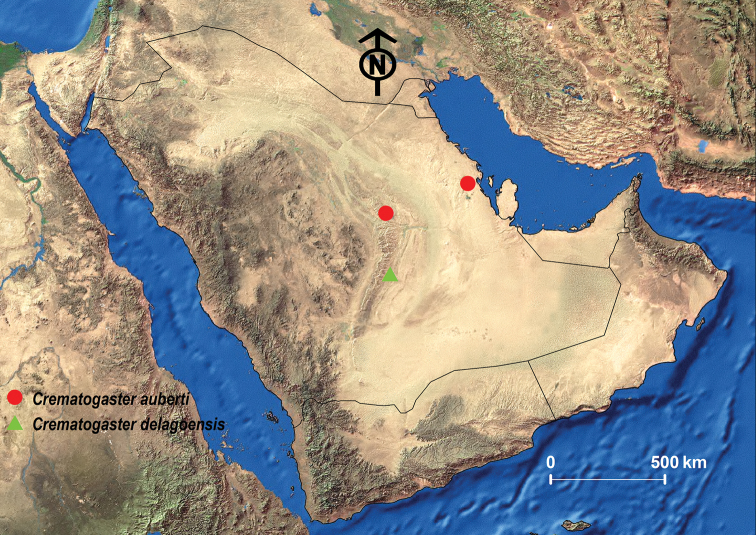
Distribution map of *C. auberti* and *C. delagoensis*.

#### Crematogaster chiarinii

Taxon classificationAnimaliaHymenopteraFormicidae

Emery

3CA7ED7C-C29F-5251-8D1B-6C1026B38A3B

Figure 12A–C

##### Taxonomic history.

*Crematogaster chiarinii* Emery, 1881: 271, fig. (w.) Ethiopia: Forel 1892: 353 (q., m.).

Combination in Crematogaster (Crematogaster): Wheeler 1922: 835; in Crematogaster (Acrocoelia): Emery 1922: 146; in Crematogaster (Crematogaster): Bolton 1995: 166.

Current subspecies: *C. chiarinii aethiops*, *C. chiarinii bayeri*, *C. chiarinii cincta*, *C. chiarinii nigra*, *C. chiarinii sellula*, *C. chiarinii subsulcata*, *C. chiarinii taediosa*, *C. chiarinii v-nigra*.

Crematogaster chiarinii var. affabilis Forel, 1907b: 142 (w.) Somalia. Syn. nov.

Combination in Crematogaster (Acrocoelia): Emery 1922: 146; in Crematogaster (Crematogaster): Bolton 1995: 166.

##### Material examined.

**KSA**: Al Baha Province: Elqamh park, Baljurshi, 19.913056N, 41.905E, 1931 m, 17.v.2010 (Sharaf MR) (12 w, KSMA); Al Mukhwah, Dhi Ayn village, 19.929417N, 41.441722E, 741 m, 18.v.2011 (Sharaf MR) (19 w, KSMA); Al Mukhwah, Dhi Ayn village, 19.929417N, 41.441722E, 741 m, 15.v.2011 (Sharaf MR) (4 w, KSMA); W. Elzaraeb (Kheir), 20.073417N, 41.38675E, 2123 m, 15.v.2010 (Sharaf MR) (3 w, KSMA); Amadan, Almandaq, 20.245278N, 41.468333E, 1881 m, 19.v.2010 (Sharaf MR) (3 w, KSMA); Al Baha city, 20.014533N, 41.4674E, 2172 m, 16.v.2010 (Sharaf MR) (3 w, KSMA); W. Jallah, 20.134472N, 41.342889E, 1912 m, 16.v.2011 (Sharaf MR) (15 w, 6 q, 6 m, KSMA); AlUrdyia gov., W. Gonouna, 19.429361N, 41.605028E, 353 m, 12.v.2011 (Sharaf MR) (3 w, KSMA); W. Turabah, Almandaq, 20.211028N, 41.288222E, 1793 m, 10.v.2011 (Sharaf MR) (21 w, KSMA); W. Turabah, Almandaq, 20.241917N, 41.262833E, 1751 m, 19.ix.2011 (Sharaf MR) (1 w with broken waist and gaster, KSMA); Baljurshi forest, 19.80559N, 41.71198E, 1930 m, 21.ix.2011 (FA Esteve) (2 w, KSMA); AlTawla (Al Baha-Taif RD), W. Bawah, 20.7496N, 41.247433E, 1310 m, 08.xi.2012 (Sharaf MR) (10 w, KSMA); W. Dafa near Eiban, 17.37463N, 43.07539E, 888 m, 12.xi.2012 (Sharaf MR) (7 w, KSMA); Asir province: Khamis Mushayt, W. Bishah, 18.333639N, 42.703583E, 1990 m, 27.iv.2011 (Sharaf MR) (4 w, KSMA); Abha, 18.2465N, 42.5117E, 25.iii.1983 (Collingwood CA) (9 w, WMLC); Ashaira, 21.7575N, 40.651944E, 1340 m, 14–15.ix.1980 (Collingwood CA) (1 w, WMLC); Ashaira, 21.7575N, 40.651944E, 1340m, 06–10.v.1936 (H. St. J. B. Philby, B.M.1936-405) (2 w, BMNH). Asir Province: Khamis Mushayt, W. Ben Hashbal, 18.594806N, 42.650361E, 26.iv.2011 (Sharaf MR) (18 w, KSMA); Khamis Mushayt, 18.3093N, 42.7662E, x.1997 (C. W. Mills) (2 w, WMLC); W. Saber, 20.18N, 41.1665E, 06.iii.2013 (Sharaf MR) (4 w, KSMA); Najran province: Najran city, 17.49590N, 44.12922E, 1323 m, 21.ii.2015 (S Salman) (3 w, KSMA); Najran, 17.5656N, 44.2289E, 25.iii.1983 (Collingwood CA) (2 w, WMLC); Fayfa, 17.29691N, 43.135E, iii.1983 (Collingwood CA) (25 w, WMLC). **Yemen**: Taiz (y529), 13.5776N, 44.0178E, x.1991 (A van Harten) (3 w, WMLC); W. Surdud, 15.266667N, 43.716667E, xii.1991 (A van Harten) (1 w, WMLC); Mabar, 14.7953N, 44.2905E, viii.1992 (A van Harten) (1 w, 1256, WMLC); W. Al Barahani, 13.3042N, 44.1003E, ii.1992 (M. Mahyoub) (1 w,1001, WMLC); Khazam, 13.49N, 43.84E, 1950 m, ix.1979 (B. Lanza) (2 w, WMLC); Taiz, Maagala, 13.5776N, 44.0178E, ii.1993 (A van Harten) (15 w, 983, WMLC); Rehab, 14.23N, 44.19E, iii.1992 (A van Harten) (8 w, 1088, WMLC); Rehab, 14.23N, 44.19E, iv.1993 (M. Knapp) (18 w, 1999, WMLC); Suq Bani Mansur, 14.59646N, 43.594325E, iv.1991 (M. Knapp) (10 w, 191, WMLC); Taiz, 15.266667N, 43.716667E, ii.1992 (M. Knapp) (20 w, 1006, WMLC); Zingibar, 13.135N, 45.3886E, iii.1993 (20 w, in tube, WMLC); Sana’a, 15.3694N, 44.191E, 11–18.iii.1937 (Dr. Carl Rathiens,B.M.1938-396) (1 w, BMNH); W.Aden Port,Al Huseini near Lahej, 13.0578N, 44.8833E, 29.xi.1937 (B.M. Expedition to S.W. Arabia, H. Scott & E. B. Britton. B.M.1933-246) (1 w, BMNH). **Oman**: W. Dhiyan, 19.7314N, 41.4219E, 13–14.ix.1983 (M. D. Gallagher) (1 w, WMLC); Dhofar, 17.0194N, 54.1108E, 04.ix.1985 (R. W. Whitcomb) (9 w, WMLC); Fasad (Dhofar), 18.4333N, 53.1333E, ii.1998 (M. D. Gallagher) (1 q, WMLC).

##### Geographic range.

This species was originally described from Ethiopia and is widely distributed in the Afrotropical region. It seems to be a predominantly eastern African species but is also known from Central and South Africa (Guénard et al. 2017; Janicki et al. 2017). From the Arabian Peninsula, the species has been recorded from the southwestern mountains of the KSA and Oman (Wheeler 1922; Borowiec 2014; Collingwood 1985, Collingwood and Agosti 1996) (Fig. 13).

##### Remarks.

*Crematogaster affabilis* was originally described as a variety of *C. chiarinii* but subsequently elevated to species rank by Collingwood (1985) based on head width, length of the propodeal spines, and absence of the mesonotal tubercle. During the present study the type material of *C. chiarinii* and *C. affabilis* (MHNG) were examined and detailed morphological examinations of the type material shows that both are uniformly brown with the anterior half of the cephalic surface longitudinally striated; the frontal triangle is well-defined with a distinct posterior carina running back to the posterior level of the eyes; the propodeal spines long and acute, about 1.5 × longer than their base, making an angle of about 45 with the longitudinal axis of the body in profile view; and, the mesonotum in profile descending abruptly to a deep metanotal groove. The type of *C. affabilis* is somewhat larger but not more than major workers in both species are from minor workers. Consequently, even though both taxa are outside the focal region of Arabia, on the basis of any lack of significant phenotypical differences, we propose *C. chiarinii* as a senior synonym of *C. affabilis*.

Nevertheless, despite our synonymizing of both taxa, the taxonomic condition of *C. chiarinii* is still in need of a thorough revision. The taxonomic history above with all status changes, synonymic history, and numerous still valid infraspecific taxa shows clearly the complexity of this task. Based on superficial examination of material from the Afrotropical region, we are doubtful that the material from East Africa might remain conspecific with the one from Central and South Africa. Hopefully, a future revision of the Afrotropical fauna will resolve this situation.

**Figure 12.**
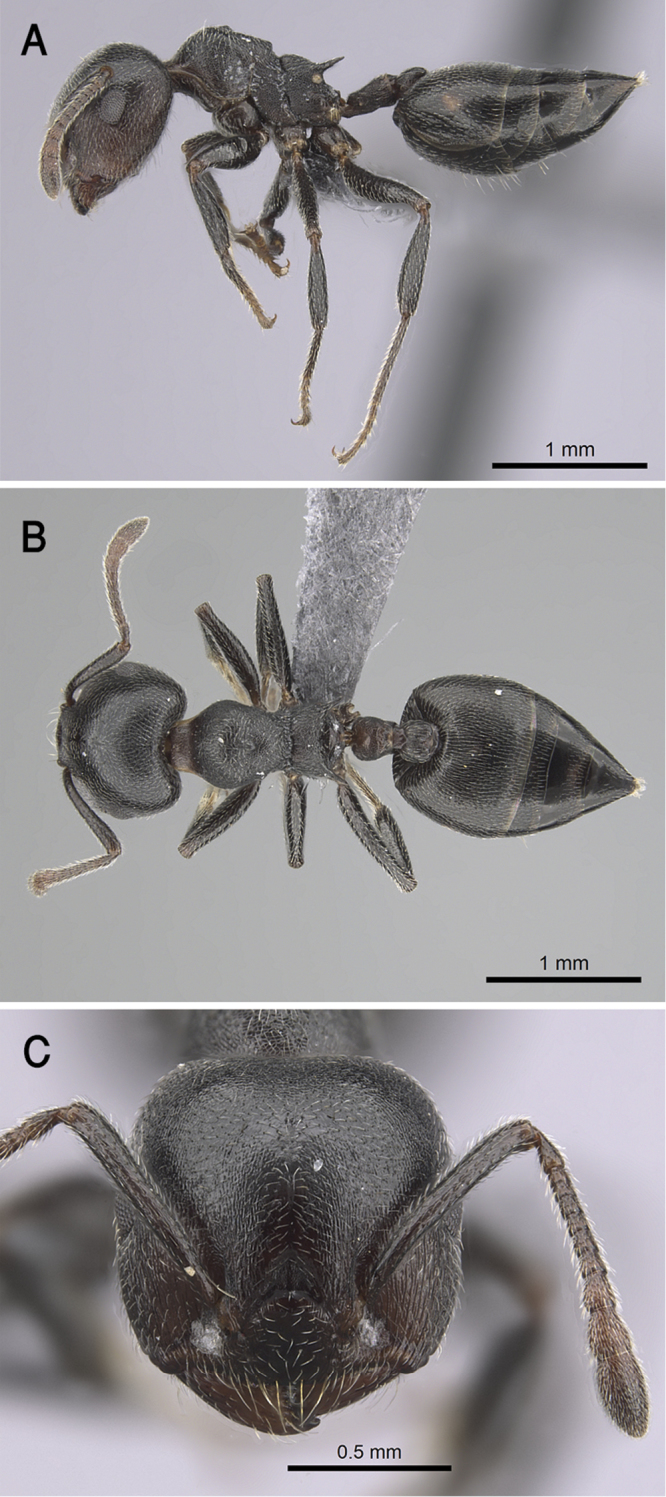
*C. chiarinii***A** body in profile **B** body in dorsal view **C** head in full-face view, CASENT0263878 (Will Ericson), www.AntWeb.org.

**Figure 13.**
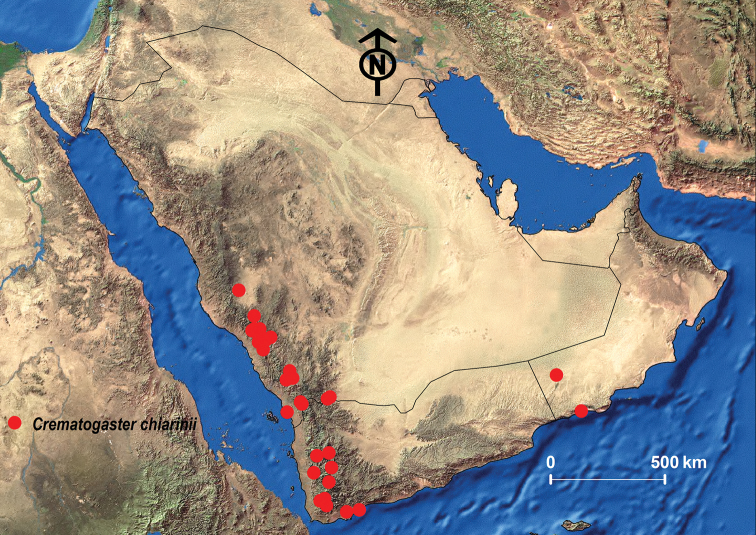
Distribution map of *C. chiarinii*.

#### Crematogaster delagoensis

Taxon classificationAnimaliaHymenopteraFormicidae

Forel

ED48585B-08F9-5290-B731-1EBB6E2D1817

Figure 14A–C

##### Taxonomic history.

Crematogaster inermis r. delagoensis Forel, 1894a: 99 (w.) Mozambique.

Combination in Crematogaster (Acrocoelia): Emery 1922: 146; in Crematogaster (Crematogaster): Bolton 1995: 166.

Status as species: Emery 1922: 146.

Current subspecies: *C. delagoensis acutidens* Arnold, *C. delagoensis merwei* Santschi, *C. delagoensis rhodesiana* Arnold.

##### Material examined.

**KSA**: Alnaifiam Farshet Shaal, 22.41559N, 46.58806E, 602 m, 12.iv.2015 (Al Dhafer et al.) (1 w, KSMA).

##### Geographic range.

This species was originally described from Mozambique and in the Afrotropical region seems to be restricted to Southern Africa (Guénard et al. 2017; Janicki et al. 2017). Our material represents the first record from the KSA since the species was previously only known from Yemen and Oman (Borowiec 2014; Collingwood and Agosti 1996; Sharaf et al. 2018) (Fig. 11).

##### Remarks.

Again, the disjunctive distribution raises some doubts about the identity of our material. Even though it is conspecific the material previously identified from Oman and Yemen, it needs to be proven that it is indeed *C. delagoensis*. The lack of records from East Africa and the southwestern parts of KSA could be due to insufficient sampling, but it could also be that the Arabian material is not similar to the South African “genuine” *C. delagoensis*.

**Figure 14.**
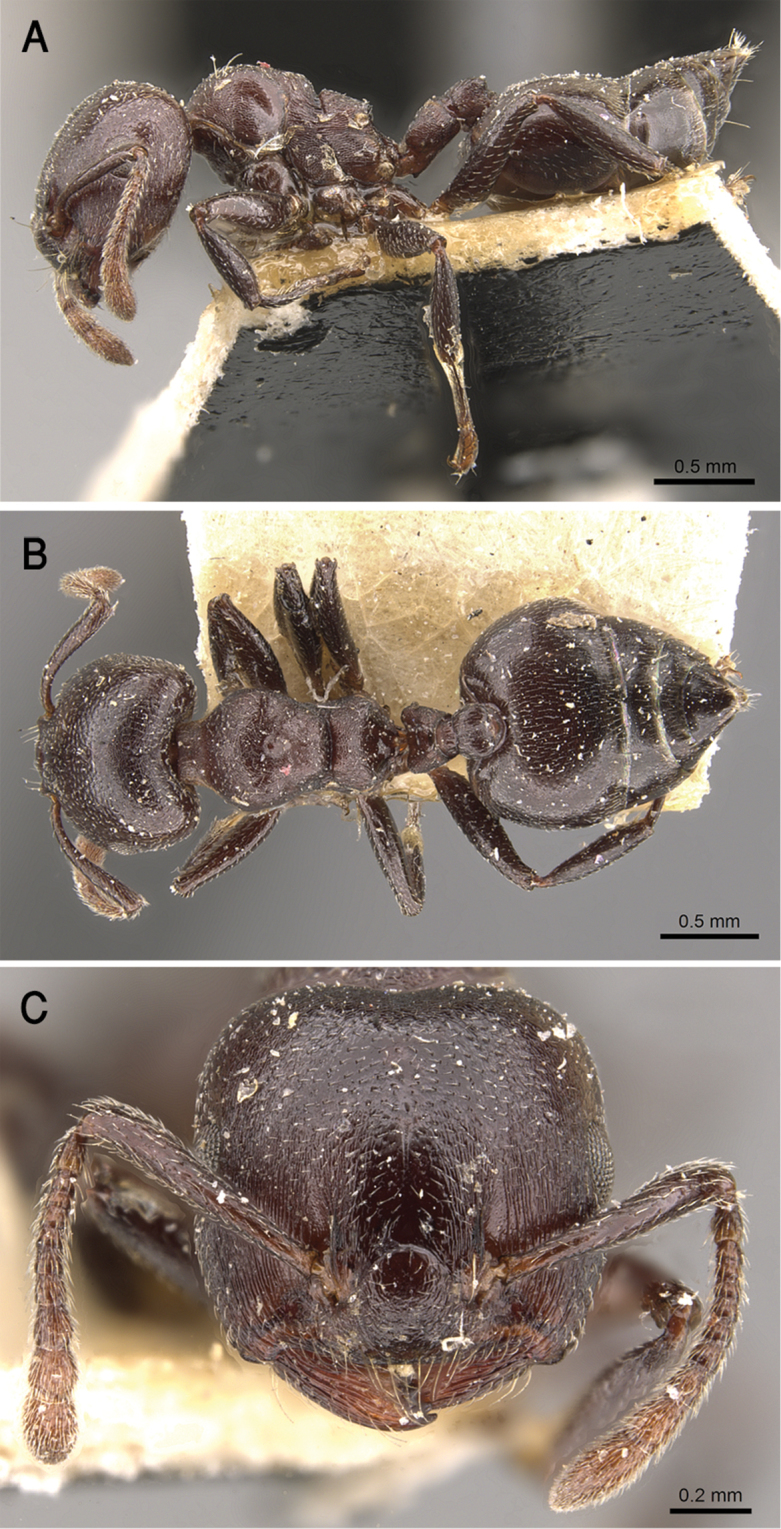
*C. delagoensis***A** body in profile **B** body in dorsal view **C** head in full-face view, CASENT0908517 (Will Ericson), www.AntWeb.org.

#### Crematogaster flaviventris

Taxon classificationAnimaliaHymenopteraFormicidae

Santschi

C697A0C3-034C-56EF-AC5B-615DD2A00778

Figure 15A–C

##### Taxonomic history.

*Crematogaster flaviventris* Santschi, 1910: 370 (w, q, m) Democratic Republic of Congo.

Subspecies of *C. inversa*: Santschi 1914a: 86; of *C. castanea*: Santschi 1935: 257.

Status as species: Collingwood and Agosti 1996: 330.

##### Geographic range.

*Crematogaster flaviventris* was originally described from the Democratic Republic of Congo and is also found in Angola, Zambia, and southern Sudan (Guénard et al. 2017; Janicki et al. 2017). On the Arabian Peninsula, it seems to be restricted to Yemen (Collingwood and Agosti 1996; Blaimer 2012a; Borowiec 2014) (Fig. 16).

##### Remarks.

This species record for Yemen is somewhat dubious. We have not examined the type material but preliminary examination of type images on AntWeb suggest that the material from Yemen is similar in color but there appear to be substantial differences in overall surface sculpture. This needs to be further investigated, ideally by comparing the Yemeni material with the type from Central Africa.

**Figure 15.**
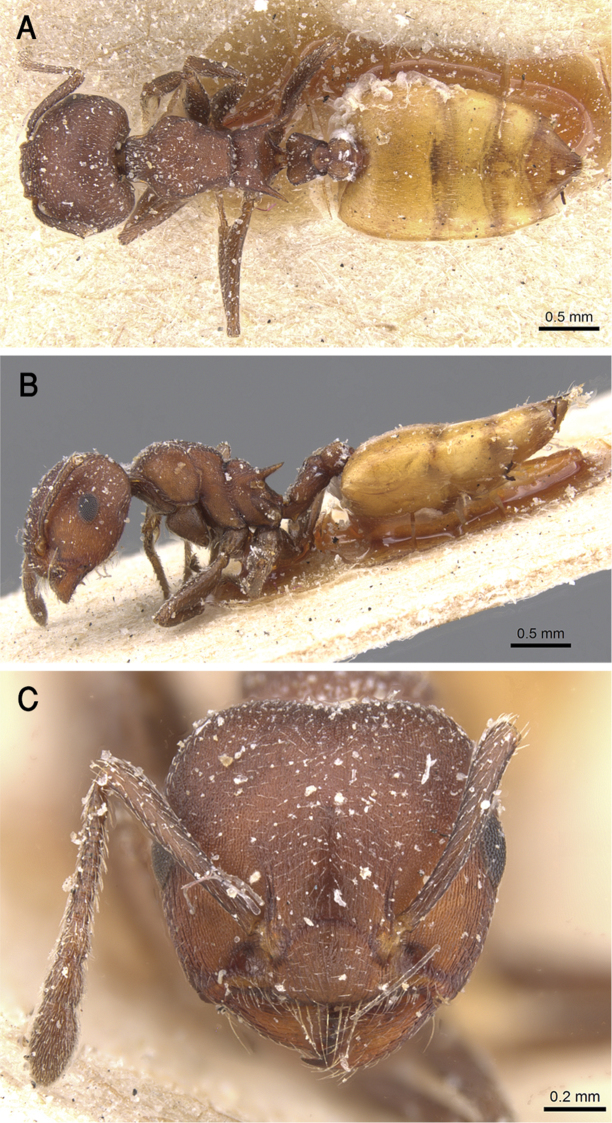
*C. flaviventris***A** body in profile **B** body in dorsal view **C** head in full-face view, CASENT0912651 (Will Ericson), www.AntWeb.org.

**Figure 16.**
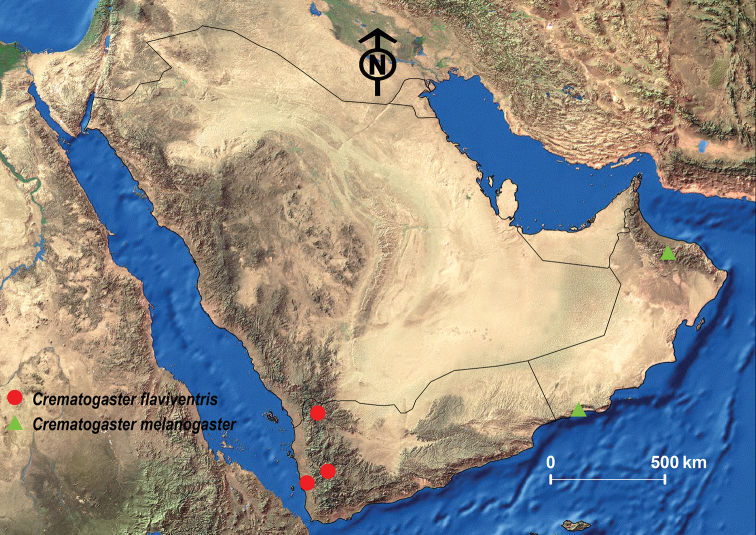
Distribution map of *C. flaviventris* and *C. melanogaster*.

#### Crematogaster gryllsi

Taxon classificationAnimaliaHymenopteraFormicidae

Sharaf & Hita Garcia sp. nov.

69051F2F-FA38-566D-A772-95D6D8013AE4

http://zoobank.org/F3844A54-2467-4CBD-A04B-5BFB079077CB

Figure 17A–C

##### Type material.

***Holotype***: pinned worker, KSA: Fayfa, 17.29691N, 43.13500E, 837 m, 06.iv.2013 (Sharaf MR) (CASENT0872096, KSMA). ***Paratypes***: 29 pinned workers, same data as the holotype (28 w, KSMA, 1 w, CASENT0919794, CASC); Asir Province, Abha, Raydah, 18.195817N, 42.389083E, 1614 m, 31.vii.2015 (Al Dhafer et al.) (8 w, KSMA; 1 w, WMLC); Abha, Raydah, 18.19465N, 42.39485E, 1851 m, 08.vi.2014 (Al Dhafer et al.) (1 w, KSMA).

##### Diagnosis.

*Crematogaster gryllsi* sp. nov. is distinguished from related congeners by the combination of the following characters: Mesonotum in profile without a tubercle on promesonotal suture; propodeal dorsum and propodeal spines forming a continuous concave curve in profile; postpetiolar node entire, not bilobed dorsally; mesopleura, petiole, and postpetiole distinctly densely punctate; color uniform yellow, second half of gaster brown-yellow.

##### Holotype worker.

EL 0.22; HL 0.75; HW 0.80; LHT 0.57; PPL 0.12; PPW 0.20; PRW 0.45; PTH 0.17; PTL 0.22; PTW 0.32; SL 0.62; ML 0.90; Indices. CI 107; LBI 158; OI 28; PPI 63; PTHI 77; PTWI 145; SI 78.

##### Paratype workers.

EL 0.15–0.25; HL 0.65–0.87; HW 0.67–0.87; LHT 0.45–0.57; PPL 0.12–0.18; PPW 0.17–0.25; PRW 0.40–0.55; PTH 0.12–0.25; PTL 0.12–0.25; PTW 0.27–0.42; SL 0.50–0.70; ML 0.75–1.0; Indices. CI 96–107; LBI 124–178; OI 22–31; PPI 59–81; PTHI 88–125; PTWI 145–267; SI 68–93 (*N* = 15).

##### Description.

***Worker*.** Workers of the new species showing marked size variation in the two nests.

*Head.* Head as long as or little broader than long with convex sides and feebly concave posterior margin; antennae 12-segmented; in full-face view antennal scapes when laid back from their insertions reach or surpass posterior margin of head by the length of the first funicular segment; eyes of moderate size (OI 22–31), located nearly at mid-length of head in full-face view and with about 12 ommatidia in the longest row; anterior clypeal margin broadly convex. *Mesosoma.* Mesonotum in profile without a small tubercle on promesonotal suture; promesonotal suture feebly impressed; mesonotum convex in profile; median mesonotal carina absent; metanotal groove shallowly impressed but distinct; propodeal spines long, sharp and upward directed; propodeal spiracle circular, and located below the base of the propodeal spines; propodeal dorsum and propodeal spines forming a continuous concave curve in profile. *Petiole.* In profile petiole distinctly longer than high (PTHI 88–125; PTWI 145–267); approx. twice broader anteriorly than posteriorly in dorsal view; subpetiolar process well developed. *Postpetiole.* Postpetiolar node entire, not bilobed in dorsal view. *Pilosity.* Cephalic and clypeal surfaces with abundant scattered fine appressed pubescence; anterior clypeal margin and mandibles with abundant scattered long yellow hairs; antennae and legs with dense appressed pubescence; mesosoma without hairs; promesonotum and mesonotum dorsum with few appressed pubescence; petiole, postpetiole and the first three gastral tergites with appressed pubescence; few hairs on the terminal gastral segment. *Sculpture.* Mandibles longitudinally striated; clypeal surface smooth; area in front of eyes finely longitudinally striated; cephalic surface smooth; antennal fossae surrounded by fine and curved striolae; promesonotum side smooth; promesonotum dorsum faintly longitudinally rugulose; propodeal dorsum faintly longitudinally striated; mesopleura, petiole, and postpetiole distinctly densely punctate; metapleura faintly, irregularly striated; gastral tergites smooth. *Color.* Head, mesosoma, petiole, postpetiole, first gastral tergite, legs, and antennae uniform yellow, second half of gaster brown-yellow.

##### Etymology.

The patronymic epithet has been selected in honor of Bear Grylls, the survival instructor in recognition of his remarkable efforts in spreading the culture of survival globally.

##### Ecological and biological notes.

The microhabitat of *Crematogaster gryllsi* in the type locality (Fayfa Mountains) (Fig. 18) is the humid leaf litter under Acacia and *Calotropis procera* (Aiton) W. T. Aiton (Apocynaceae) trees. The specimens were collected by sifting leaf litter. The material from Raydah were collected by pitfall traps.

##### Geographic range.

This new species is only known from the Asir Mountains, KSA (Fig. 19).

##### Remarks.

This distinct new species immediately be separated from all Arabian *Crematogaster* species by the undivided postpetiolar node and the uniform yellow color. All other species have a divided postpetiolar node. *Crematogaster gryllsi* appears to be related directly to *C. luctans* Forel, 1907 from Kenya as it possesses an undivided postpetiolar dorsum as well. However, *C. gryllsi* can be separated by the following characters: metanotal groove shallowly impressed, propodeal dorsum and propodeal spines forming a continuous concave curve in profile; promesonotum side smooth; mesopleura distinctly densely punctate; whereas *C. luctans* has deeply impressed, U-shaped metanotal groove profile; convex propodeal dorsum in profile extending posteriorly to straight propodeal spines; mesosomal sides longitudinally striated.

##### Comments.

The record of *C. luctans* given by Collingwood (1985) was based on material collected from Fayfa Mountains where the new species, *C. gryllsi* was collected. Considering that the shape of the postpetiole is uncommon in the Arabian fauna and the initial identification of *C. luctans* was probably based on this character, it is highly likely that the record of *C. luctans* by Collingwood (1985) represents a misidentification. This hypothesis is further supported by the complete lack of any material of *luctans* in the WMLC. Therefore, the species was omitted from the list of Arabic species.

**Figure 17.**
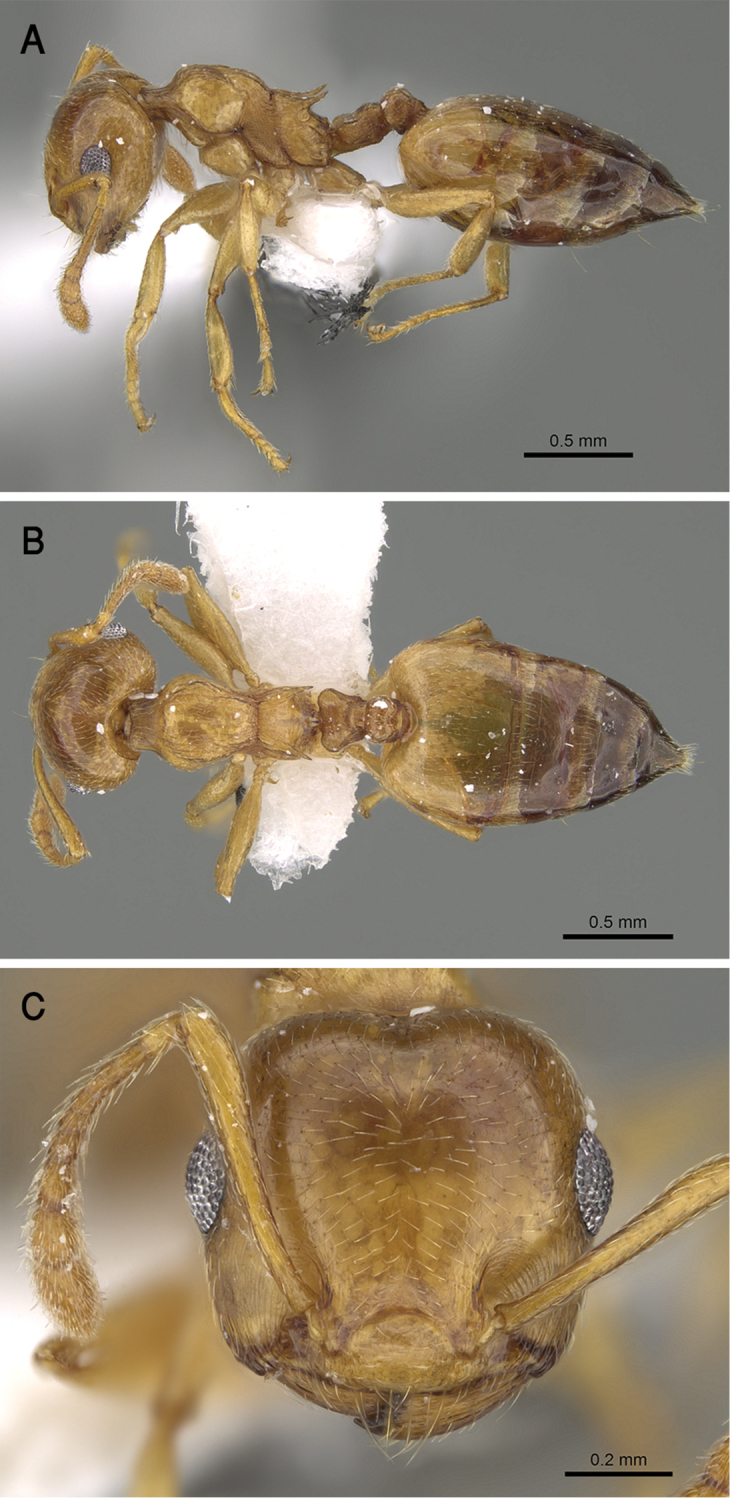
*C. gryllsi* sp. nov. **A** body in profile **B** body in dorsal view **C** head in full-face view, CASENT0919794 (Michele Esposito), www.AntWeb.org.

**Figure 18.**
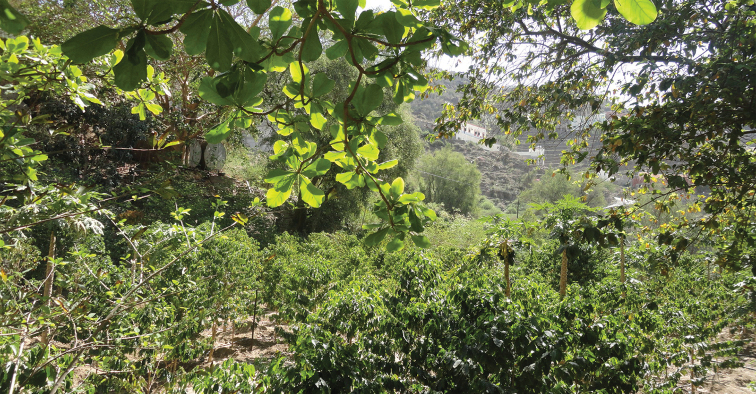
Fayfa, type locality of *C. gryllsi* sp. nov. (M. Sharaf).

**Figure 19.**
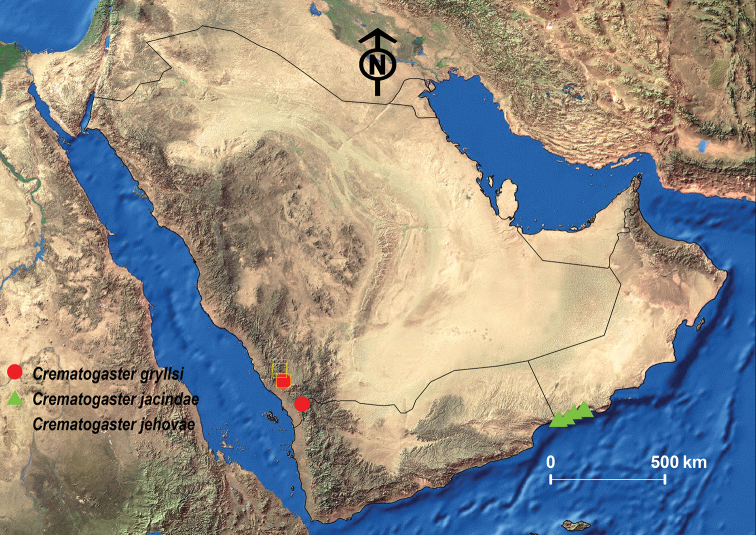
Distribution map of *C. gryllsi* sp. nov., *C. jacindae* sp. nov. and *C. jehovae*.

#### Crematogaster inermis

Taxon classificationAnimaliaHymenopteraFormicidae

Mayr

A9F86DC9-E059-550D-BDB2-E7E6293AB97B

Figure 20A–C

##### Taxonomic history.

*Crematogaster inermis* Mayr, 1862: 766 (w.) Egypt. André 1883: 395 (q.); Santschi 1937: 296 (q.); Santschi 1938: 38 (m.).

Combination in Crematogaster (Crematogaster): Wheeler 1922: 840; in Crematogaster (Acrocoelia): Emery 1922: 143; in Crematogaster (Crematogaster): Bolton 1995: 166.

Current subspecies: *C. inermis aphrodite* Santschi, *C. inermis armatula* Emery, *C. inermis lucida* Forel.

##### Material examined.

**Egypt**: Elmenia, Abu Swelam, 30.75N, 28.1E, 29.vi.2003 (Sharaf MR) (1 w, OUMC).

##### Geographic range.

*Crematogaster inermis* was originally described from Egypt and is widely distributed from the Iberian Peninsula through North Africa to the Middle East (Borowiec 2014; Guénard et al. 2017; Janicki et al. 2017). For the Arabian Peninsula, this species was only recorded from Yemen (Collingwood and van Harten 2001; Borowiec 20114).

##### Remarks.

Knowing that the species is widespread in the Mediterranean and Middle East and the only record for the Arabian Peninsula is from Yemen, we think that it is likely that it is also present in the KSA. Futher sampling is necessary to verify this.

**Figure 20.**
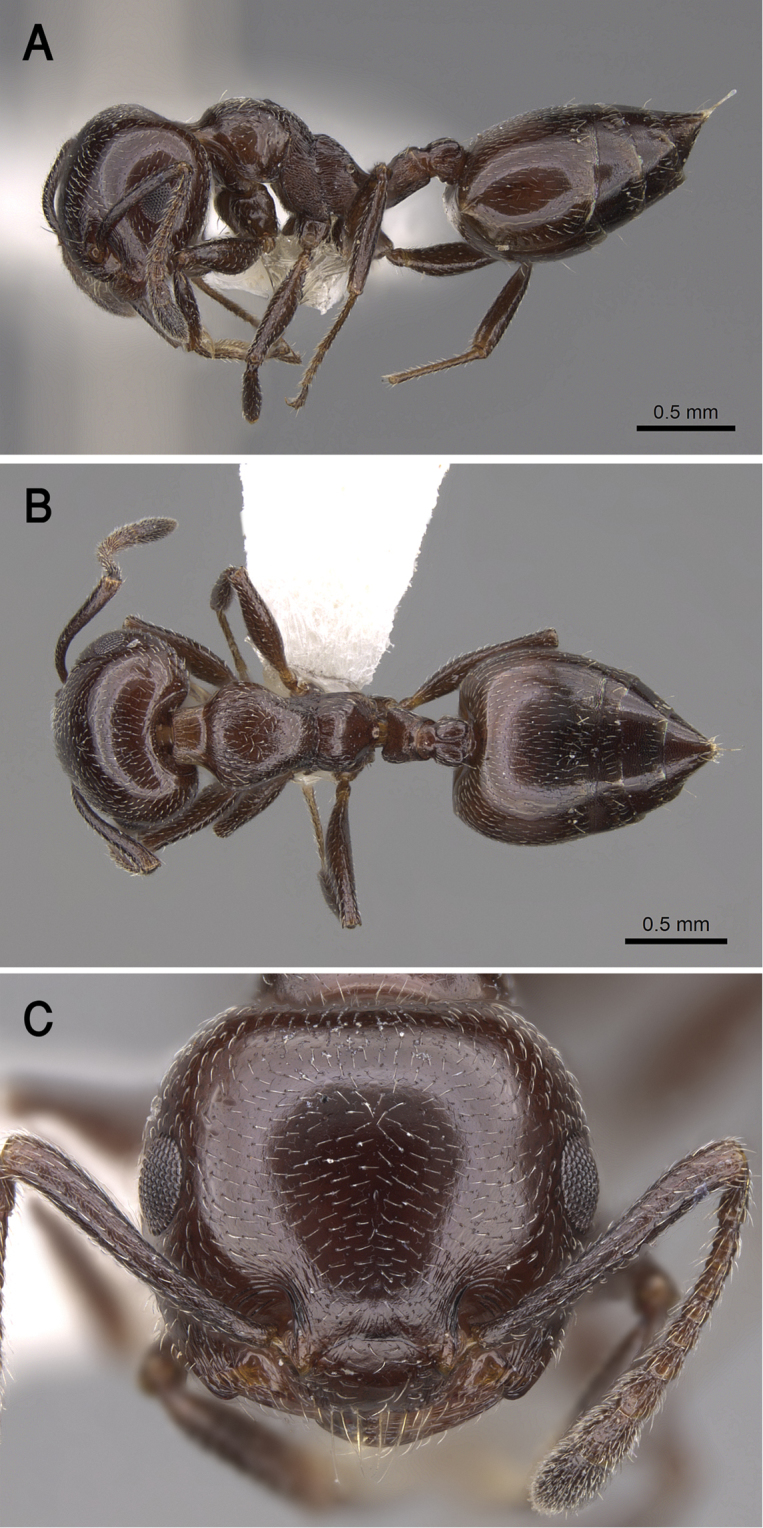
*C. inermis***A** body in profile **B** body in dorsal view **C** head in full-face view, CASENT0922679 (Wade Lee), www.AntWeb.org.

#### Crematogaster jacindae

Taxon classificationAnimaliaHymenopteraFormicidae

Sharaf & Hita Garcia sp. nov.

D25340C6-4E5D-535B-BE2D-926632673443

http://zoobank.org/15D56546-DEA0-4764-AA6C-D2BADC5A4621

Figure 21A–C

##### Type material.

***Holotype***: pinned worker, Oman: Dhofar, Ayn Sahlanoot, 17.14766N, 54.17878E, 151 m, 16.xi.2017 (Sharaf MR) (CASENT0872068, KSMA). ***Paratype***: pinned workers, Oman: Dhofar: Ayn Hamran, 17.08631N, 54.28043E, 56 m, 22.xi.2017, BS (Sharaf MR) (11 w, KSMA, 1 w, CASENT0922856, CASC, 1 w, WMLC); Ayn Razat, 17.12443N, 54.23832E, 98 m, 20.xi.2017, ML (Sharaf MR) (8 w); Dhalkout, 16.72673N, 53.24942E, 623 m, 18.xi.2017, SF (Sharaf MR) (16 w); Dhalkout, 16.70703N, 53.25068E, 34 m, 19.xi.2017, BS (Sharaf MR) (7 w); Dhalkout, 16.69273N, 53.15621E, 628 m, 18.xi.2017, BS (Sharaf MR) (17 w); Ayn Sahlanot, 17.14766N, 54.17878E, 151 m, 16.xi.2017, BS (Sharaf MR) (4 w); Ayn Ashat, 16.99810N, 53.81954E, 202 m, 21.xi.2017, SF (Sharaf MR) (20 w); Dhalkout road, Aghbaroot village, 16.79818N, 53.55392E, 1034 m, 18.xi.2017, BS (Sharaf MR) (1 w) (KSMA).

##### Diagnosis.

*Crematogaster jacindae* sp. nov. is distinguished from related congeners by the combination of the following characters: median mesonotal carina absent; propodeal spines absent; propodeal spiracle distinct in the form of a slit; area in front of eyes finely longitudinally striated; antennal fossae surrounded by fine and curved striolae; head black-brown or black, mesosoma, petiole and postpetiole dark brown, relatively lighter than head, gaster golden yellow.

##### Holotype worker.

EL 0.20; HL 0.75; HW 0.82; LHT 0.55; PPL 0.12; PPW 0.20; PRW 0.42; PTH 0.12; PTL 0.25; PTW 0.22; SL 0.55; ML 0.87; Indices. CI 109; LBI 158; OI 24; PPI 167; PTHI 48; PTWI 88; SI 67.

##### Paratype workers.

EL 0.17–0.22; HL 0.72–0.92; HW 0.75–1.0; LHT 0.52–0.75; PPL 0.12–0.17; PPW 0.15–0.25; PRW 0.32–0.50; PTH 0.12–0.17; PTL 0.20–0.32; PTW 0.20–0.30; SL 0.50–0.95; ML 0.77–1.02; Indices. CI 96–113; LBI 121–165; OI 16–27; PPI 125–208; PTHI 44–68; PTWI 78–125; SI 61–95 (*N* = 20)

##### Description.

***Worker*.***Head.* Head as long as or little broader than long with convex sides and feebly concave posterior margin; antennae 12-segmented; in full-face view antennal scapes when laid back from their insertions fail to reach posterior margin of head; eyes of moderate size (OI 16–27), located nearly at mid-length of head in full-face view and with ca. eleven ommatidia in the longest row; anterior clypeal margin broadly convex. *Mesosoma.* Promesonotum and mesonotum forming continuous curve in profile; median mesonotal carina absent; metanotal groove well developed; propodeal dorsum short forming curve with longer propodeal declivity; propodeal spines absent; propodeal spiracle distinct and slit-shaped. *Petiole.* In profile petiole distinctly longer than high (PTHI 44–68; PTWI 78–125); broader anteriorly than posteriorly in dorsal view. *Postpetiole.* Postpetiolar node distinctly bilobed in dorsal view; nearly as high as petiole in profile. *Pilosity.* Cephalic surface with abundant scattered fine pale hairs; anterior clypeal margin and mandibles with several long yellow hairs; posterior half of clypeus without hairs or pubescence; antennae and legs with abundant appressed pubescence; promesonotum and mesonotum each with single pair of hairs; promesonotum and mesonotum dorsum with appressed pale pubescence; no hairs or pubescence on propodeum; petiole and postpetiole each with single pair of posteriorly directed hairs; gastral pilosity restricted to few pairs on posterior margins of gastral tergites; gastral tergites with scattered appressed pubescence. *Sculpture.* Mandibles longitudinally striated; clypeal surface smooth; area in front of eyes finely longitudinally striated; cephalic surface feebly imbricate; antennal fossae surrounded by fine and curved striolae; promesonotum lateral side faintly imbricate; promesonotum dorsum faintly reticulate rugulose; mesopleura, metapleura, petiole, and postpetiole distinctly densely imbricate; gastral tergites faintly imbricate. *Color.* Head black-brown or black, mesosoma, petiole and postpetiole dark brown, relatively lighter than head, gaster golden yellow and strongly contrasting with remainder of body.

##### Etymology.

The patronymic epithet has been selected in honor of Ms. Jacinda Ardern, the Prime Minister of New Zealand in recognition of her humanitarian attitudes towards Muslim and minority communities in New Zealand.

##### Ecological and biological notes.

The microhabitats where *C. jacindae* sp. nov. was encountered included leaf litter, soil, under stones, or on native vegetation, especially acacia trees (Fig. 22). The majority of specimens were collected foraging on plants using a beating sheet, but workers were also observed foraging on the ground and wild shrubs.

##### Geographic range.

This new species is only known from Oman (Fig. 19).

##### Remarks.

The distinctive golden yellow gaster and complete lack of propodeal armament of *C. jacindae* sp. nov. allows this Omani species to be immediately recognized from all other Arabian species. The closest relative of the new species is *Crematogaster inermis* Mayr, 1862 from Egypt. Both species are similar in body size and the lack of propodeal spines, but *C. jacindae* sp. nov. can be readily separated by the following characters: area in front of eyes finely longitudinally striated; cephalic surface feebly imbricate; eyes with about 11 ommatidia in the longest row; posterior half of clypeus without hairs or pubescence; mesopleura and metapleura distinctly densely imbricate; mesonotum with a single pair of hairs and without anterior tubercles; propodeal spiracles distinct in the form of a slit; body bicolored with head black-brown or black, mesosoma, petiole and postpetiole dark brown, relatively lighter than head, gaster golden yellow. By contrast, *C. inermis* has an unsculptured cephalic surface including the area in front of the eyes, eyes with ca. 14 ommatidia in the longest row, the posterior half of clypeus with fine appressed pubescence, mesosoma with a small anterior tubercle close to the promesonotal suture seen in profile; mesopleura and metapleura longitudinally striated, mesonotum without hairs, propodeal spiracle circular, unicolorous black-brown body.

**Figure 21.**
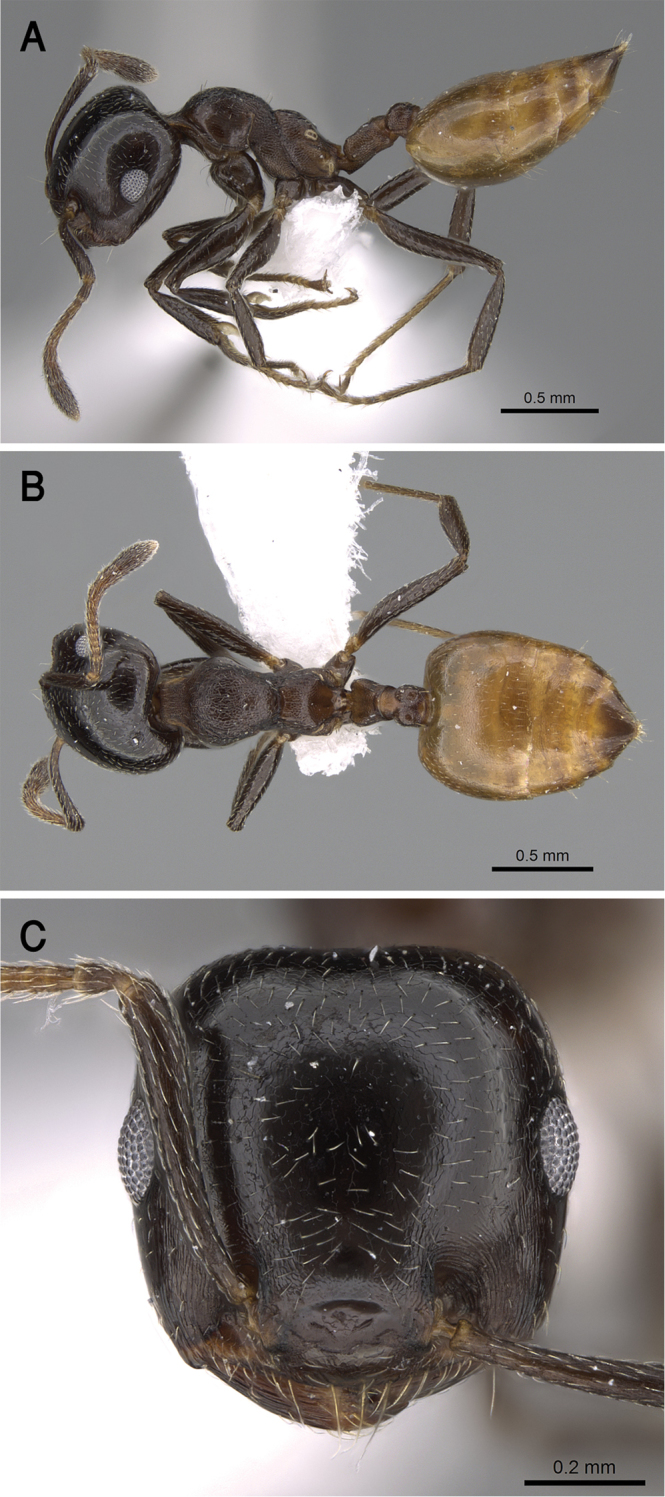
*C. jacindae* sp. nov., paratype worker **A** body in profile **B** body in dorsal view **C** head in full-face view, CASENT0922856 (Michele Esposito), www.AntWeb.org.

**Figure 22.**
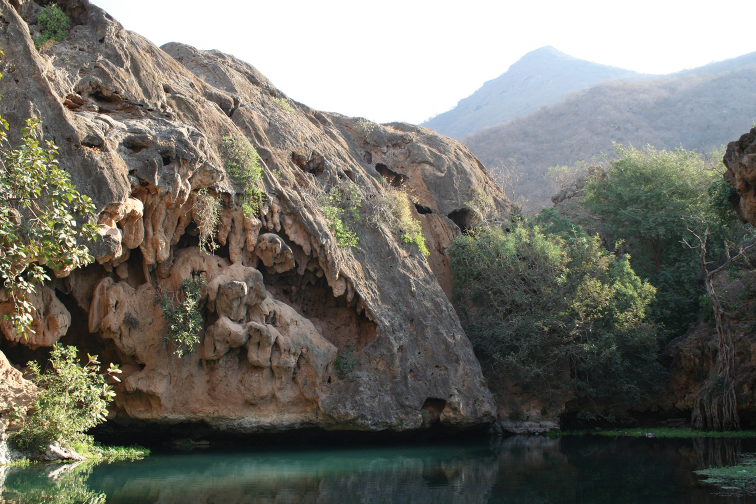
Ayn Sahlanoot, Dhofar, type locality of *C. jacindae* sp. nov. (photo: Annette Patzelt).

#### Crematogaster jehovae

Taxon classificationAnimaliaHymenopteraFormicidae

Forel

EE794796-FF87-506B-8825-42076D47FD3E

Figure 23A–C

##### Taxonomic history.

Crematogaster (Acrocoelia) auberti subsp. jehovae Forel, 1907c: 207 (w.) Israel: Menozzi 1933: 58 (q.).

Combination in Crematogaster (Acrocoelia): Emery 1922: 142; Santschi 1930: 266; in Crematogaster (Crematogaster): Bolton 1995: 166.

Status as species: Emery 1926: 3; Menozzi 1933: 58.

Current subspecies: *C. jehovae crawleyi* Emery.

##### Material examined.

**KSA**: Asir province, Ballasmer, A’l Omer, 18.76008N, 42.26806E, 2455 m, 28.iv.2019 (Sharaf MR) (1 w, KSMA); Raydah: 18.204267N, 42.4124E, 2820 m, 21.ii.2014 (Al Dhafer et al.) (4 w, KSMA); Raydah: 18.20525N, 42.410117E, 2761 m, 21.ii.2014 (Al Dhafer et al.) (1 w, KSMA); Ballasmer, A’l Azza, 18.60815N, 42.24628E, 2611 m, 27.iv.2019 (Sharaf MR) (2 w, KSMA).

##### Geographic range.

This species was originally described from Israel and is also known from the southeastern Europe and the Middle East (Borowiec and Salata 2012; Borowiec 2014; Guénard et al. 2017; Janicki et al. 2017). Our material represents the first record from the KSA since it was previously only known from UAE and Yemen (Borowiec and Salata 2012; Borowiec 2014) (Fig. 19).

##### Remarks.

Considering that the species was known from most countries around the KSA, it is not surprising to now be discovered in this region. The taxonomy and identification of this species seems straightforward and we expect that this will remain this way.

**Figure 23.**
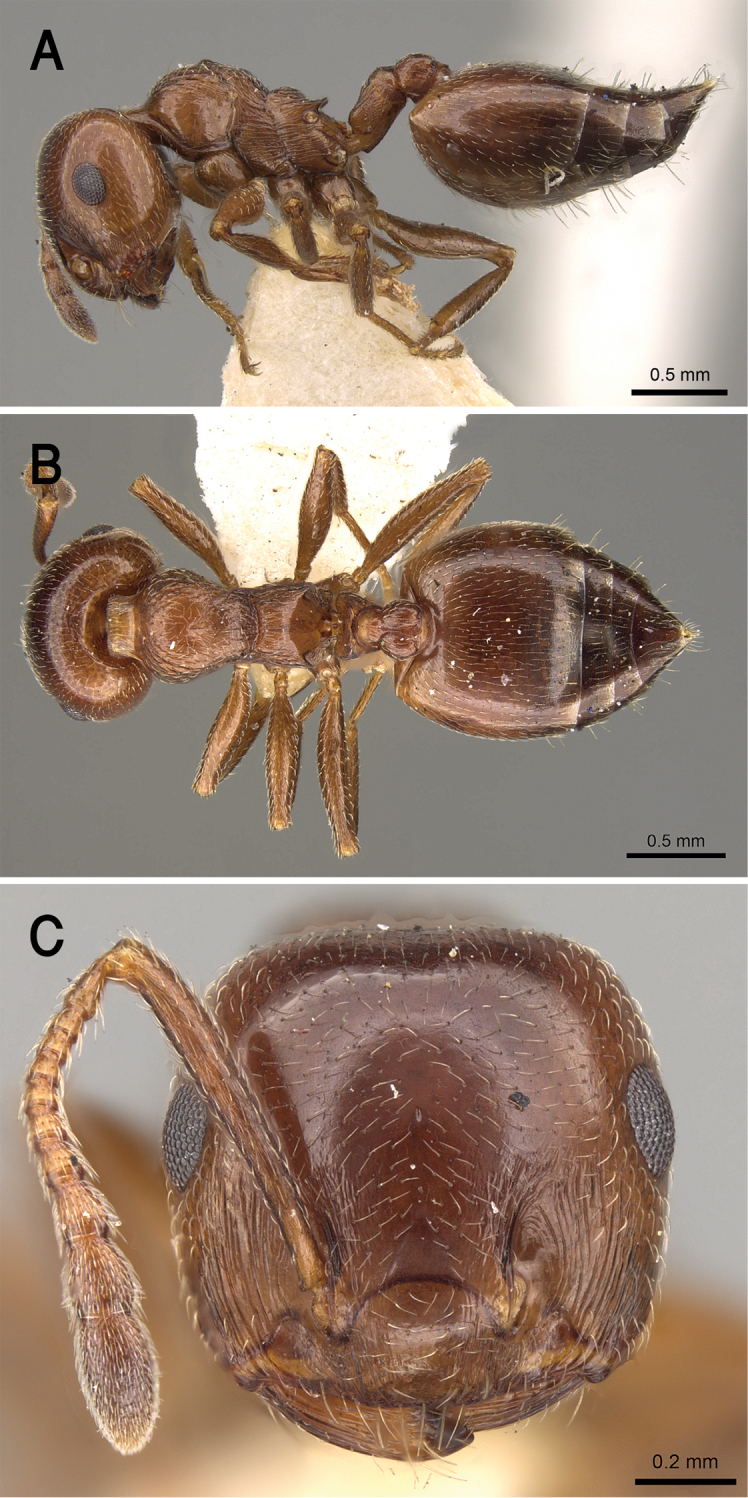
*C. jehovae***A** body in profile **B** body in dorsal view **C** head in full-face view, CASENT0914150 (Zach Lieberman), www.AntWeb.org.

#### Crematogaster laestrygon

Taxon classificationAnimaliaHymenopteraFormicidae

Emery

FA049D80-64E6-58BA-A429-25CA86ED5C1B

Figure 24A–C

##### Taxonomic history.

*Crematogaster laestrygon* Emery, 1869b: 135 (w.) Italy: De Stefani 1889: 145 (q.); Baroni Urbani 1964: 43 (q.).

Combination in Crematogaster (Acrocoelia): Emery 1922: 142; in Crematogaster (Crematogaster): Bolton 1995: 166.

Subspecies of *C. scutellaris*: Emery and Forel 1879: 464; André 1883: 393; of *C. schmidti*: Emery 1891: 15; of *C. auberti*: Forel 1902: 154; Ruzsky 1902: 31; Stitz 1917: 341; Santschi 1921: 70; Emery 1922: 142; Santschi 1934: 167.

Status as species: Forel 1909: 104; Santschi 1915: 59; Emery 1926: 2; Wheeler 1927: 106; Santschi 1936: 201.

Current subspecies: *C. laestrygon airensis* Santschi, *C. laestrygon atlantis* Santschi, *C. laestrygon canariensis* Barquín, *C. laestrygon diminuta* Santschi, *C. laestrygon granulata* Santschi, *C. laestrygon maura* Forel, *C. laestrygon submaura* Lomnicki, *C. laestrygon theryi* Santschi, *C. laestrygon vivax* Santschi.

Crematogaster laestrygon subsp. vaucheri Emery, 1926: 2 (w.) Morocco.

[First available use of Crematogaster auberti st. laestrygon var. vaucheri Santschi, 1921: 71; unavailable name.] Junior synonym of *C. laestrygon*: Collingwood 1978: 69.

##### Material examined.

**Yemen**: Hada, Sana’a, 15.3N, 44.166667E, 15.vii.1988 (P. Haney) (1 w, CASC).

##### Geographic range.

This species is originally described from Italy and widespread in southern Europe, North Africa and the Middle East (Borowiec 2014; Guénard et al. 2017; Janicki et al. 2017). The Arabian records are from the KSA and Yemen (Borowiec 2014; Collingwood 1985, Collingwood and Agosti 1996) (Fig. 25).

##### Remarks.

This is a good example of a *Crematogaster* species with a complex and uncertain taxonomic situation. It is relatively widespread in the Mediterranean and Middle East and currently has nine subspecies. It is doubtful that they are conspecific and it is possible that this is a species complex. As outlined below, we consider *C. laestrygon striaticeps* as sufficiently different to raise it to the rank of species.

**Figure 24.**
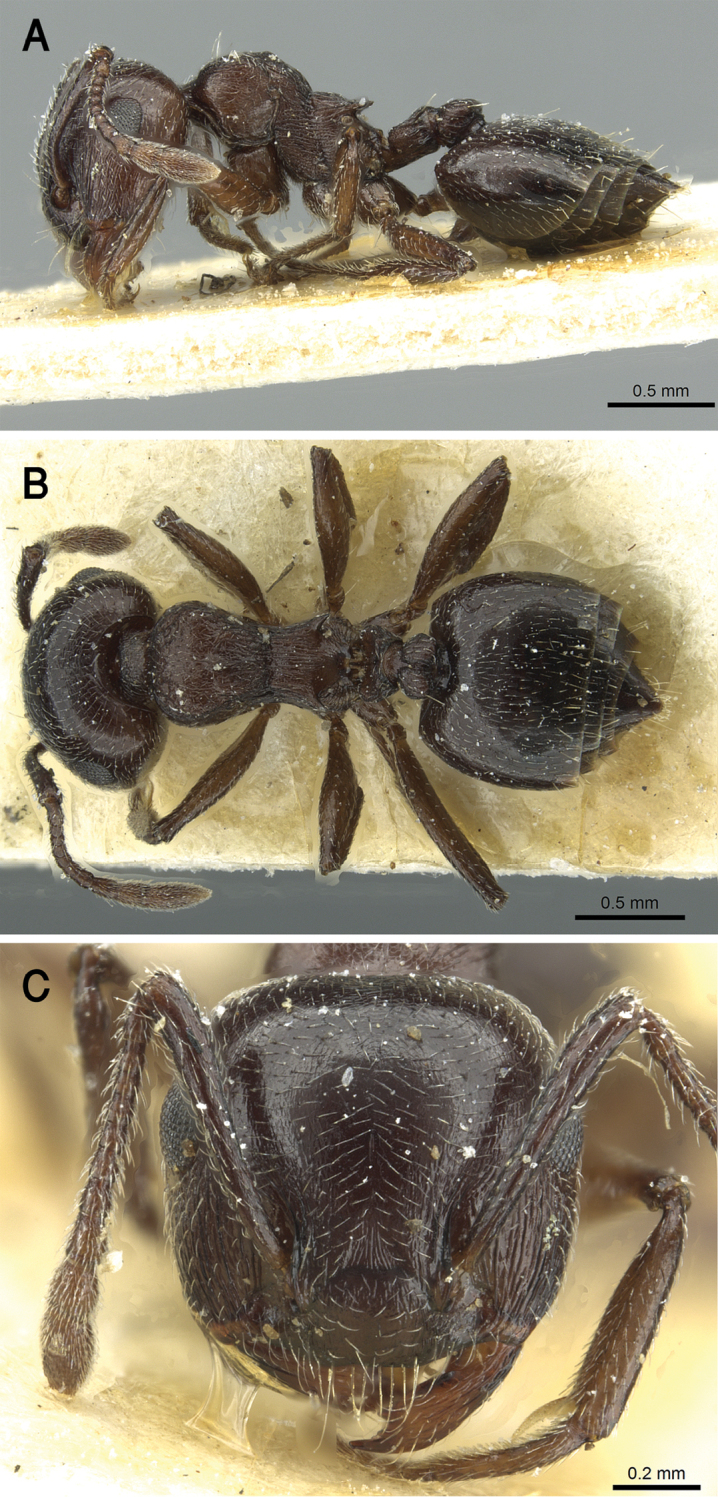
*C. laestrygon***A** body in profile **B** body in dorsal view **C** head in full-face view, CASENT0912691 (Zach Lieberman), www.AntWeb.org.

**Figure 25.**
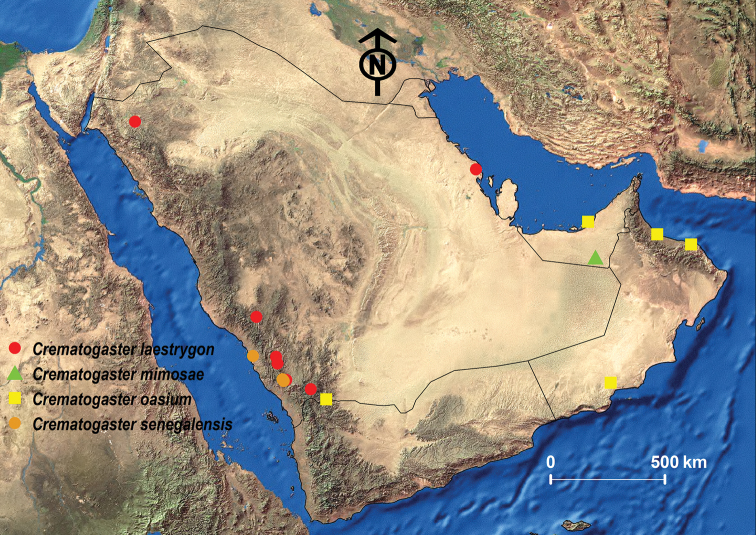
Distribution map of *C. laestrygon*, *C. mimosae*, *C. oasium*, and *C. senegalensis*.

#### Crematogaster melanogaster

Taxon classificationAnimaliaHymenopteraFormicidae

Emery

17912BBB-540B-5B8C-AE28-58E3FCD35DA2

Figure 26A–C

##### Taxonomic history.

Crematogaster arborea subsp. melanogaster Emery, 1895: 29 (w., q.) South Africa.

Combination in Crematogaster (Crematogaster): Wheeler 1922: 840; in Crematogaster (Acrocoelia): Emery 1922: 147; in Crematogaster (Crematogaster): Bolton 1995: 166.

Status as species: Wheeler 1922: 840.

Current subspecies: *C. melanogaster homonyma* Emery.

##### Geographic range.

This species was described from South Africa for the Afrotropical region and it seems to be restricted to the southern African countries of Botswana, Namibia, and South Africa (Guénard et al. 2017; Janicki et al. 2017). The only records for the Arabian Peninsula are from Oman (Collingwood and Agosti 1996; Borowiec 2014; Sharaf et al. 2018) (Fig. 16).

##### Remarks.

The species record from Oman appears doubtful based on the strange distribution pattern noted above. However, since we were unable to examine any material of this species, we consider it prudent to list it as an Arabian species for the moment.

**Figure 26.**
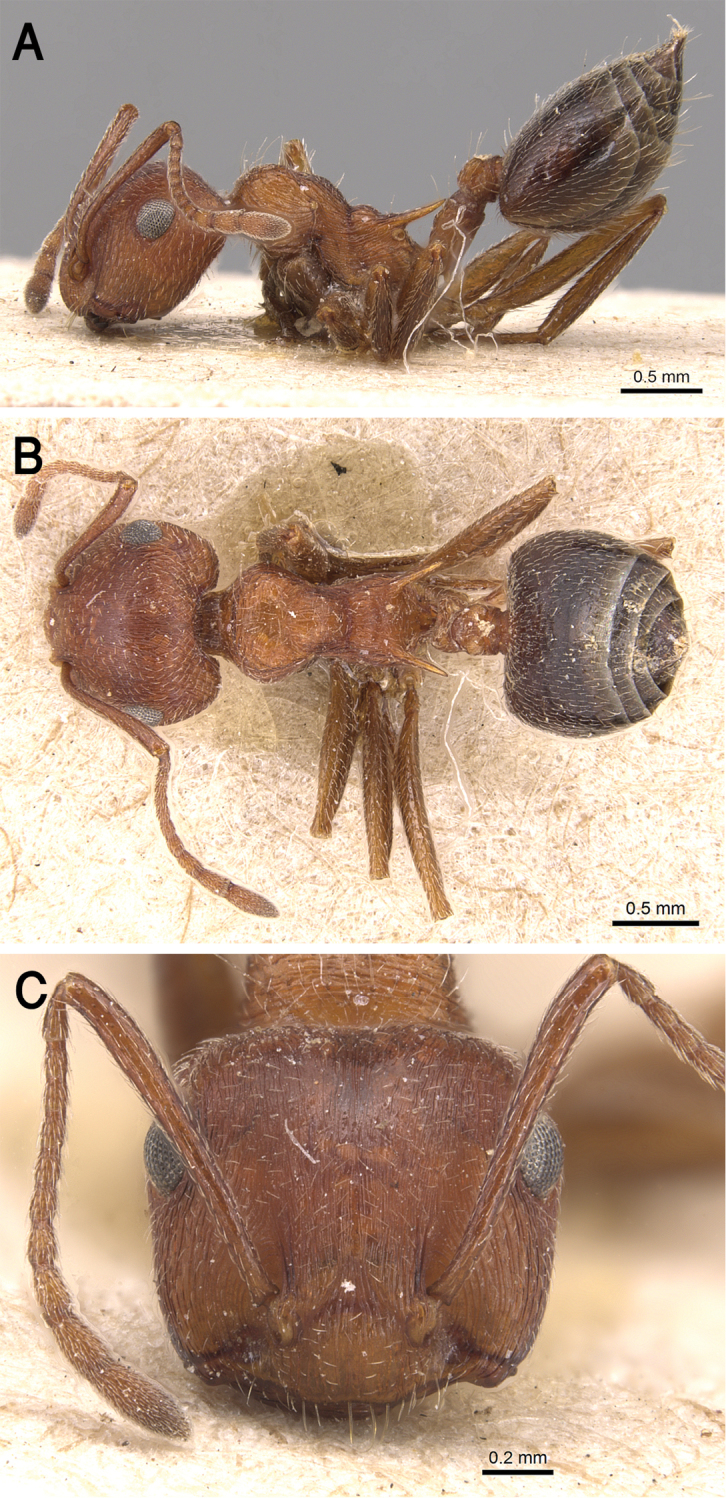
*C. melanogaster***A** body in profile **B** body in dorsal view **C** head in full-face view, CASENT0904511 (Will Ericson), www.AntWeb.org.

#### Crematogaster mimosae

Taxon classificationAnimaliaHymenopteraFormicidae

Santschi

E8FAF849-0321-5858-8694-7962BD29E469

Figure 27A–C

##### Taxonomic history.

*Crematogaster mimosae* Santschi, 1914a: 87, fig. 11 (w.) Kenya: Menozzi 1939: 105 (q.).

Combination in Crematogaster (Crematogaster): Wheeler 1922: 841; in Crematogaster (Acrocoelia): Emery 1922: 148; in Crematogaster (Crematogaster): Bolton 1995: 166.

Current subspecies: *C. mimosae tenuipilis* Santschi.

##### Geographic range.

Initially described from Kenya, in the Afrotropics this species is East African in its distribution found in Kenya, Uganda, Somalia, Sudan, and Tanzania (Guénard et al. 2017; Janicki et al. 2017). In the Arabian Peninsula, it was recorded from the KSA, Oman, the UAE and Yemen (Collingwood 1985, Collingwood and Agosti 1996; Borowiec 2014; Sharaf et al. 2018) (Fig. 25).

##### Remarks.

*Crematogaster mimosae* is one of four species of obligate acacia ants, which have been well studied in East Africa, mostly Kenya (e.g., Young et al. 1997; Martins 2010). From a taxonomic perspective, this is one of the “easy” cases within the genus in Arabia, thus very straightforwardly identifiable.

**Figure 27.**
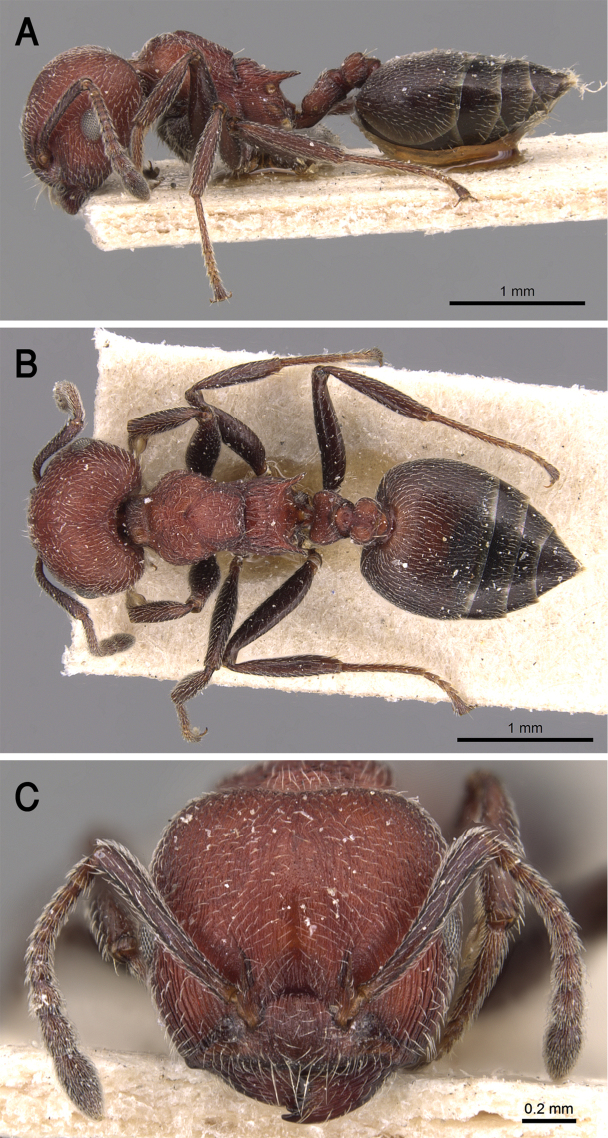
*C. mimosae***A** body in profile **B** body in dorsal view **C** head in full-face view, CASENT0904507 (Will Ericson), www.AntWeb.org.

#### Crematogaster oasium

Taxon classificationAnimaliaHymenopteraFormicidae

Santschi

72AC2D2E-CCF5-56D5-84F8-EAC2D8F236AF

Figure 28A–C

##### Taxonomic history.

Crematogaster (Acrocoelia) auberti st. oasium Santschi, 1911: 84 (w.) Tunisia; Santschi 1937: 303 (q.); Bernard 1953: 156 (m.).

Combination in Crematogaster (Acrocoelia): Emery 1922: 142; in Crematogaster (Crematogaster): Bolton 1995: 166.

Subspecies of *C. antaris*: Santschi 1921: 71; of *C. auberti*: Santschi 1929: 99.

Status as species: Santschi 1937: 303; Collingwood 1985: 261.

Current subspecies: *C. oasium saharensis* Santschi.

##### Geographic range.

*Crematogaster oasium* was described from Tunisia and can be found from Morocco east to Egypt (Borowiec 2014; Guénard et al. 2017; Janicki et al. 2017). In the Arabian Peninsula, it was recorded from Kuwait, KSA, Oman, and UAE (Collingwood 1985, Collingwood and Agosti 1996; Collingwood et al. 2011; Borowiec 2014; Sharaf et al. 2018) (Fig. 25).

##### Remarks.

This species seems to be common and widespread in northern Africa. Since we have not collected or examined any material from Arabia we list this provisionally as an Arabian species for the time being since it was listed by several authors (see above).

**Figure 28.**
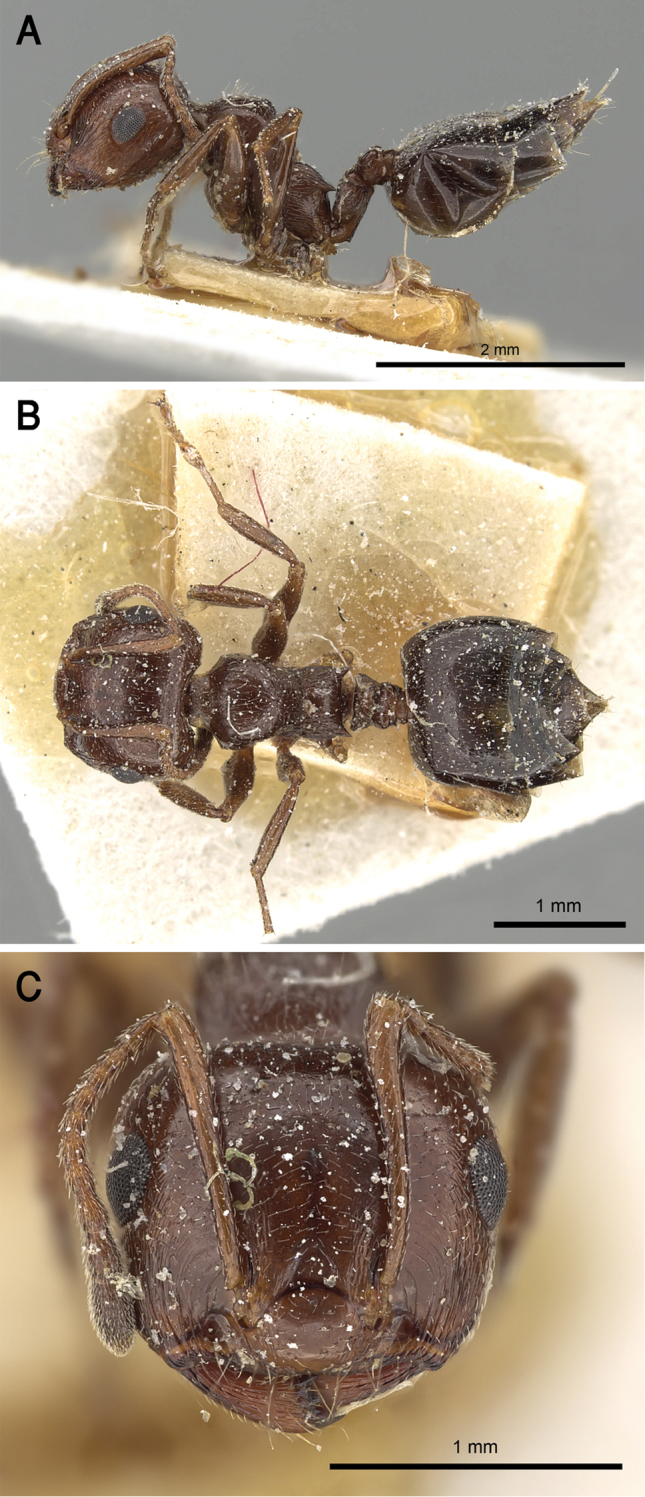
*C. oasium***A** body in profile **B** body in dorsal view **C** head in full-face view, CASENT0249821 (Ryan Perry), www.AntWeb.org.

#### Crematogaster senegalensis

Taxon classificationAnimaliaHymenopteraFormicidae

Roger

572ED382-90D9-5897-9711-C4AB99F7A37E

Figure 29A–C

##### Taxonomic history.

*Crematogaster senegalensis* Roger, 1863: 206 (w., q.) Senegal.

Combination in Crematogaster (Acrocoelia): Emery 1922: 144; in Crematogaster (Crematogaster): Bolton 1995: 166.

Subspecies of *C. aegyptiaca*: Santschi 1914b: 344; Emery 1915: 10; Wheeler 1922: 828; Emery 1922: 144; Santschi 1929: 148.

Status as species: Forel 1922: 93; Bernard 1950: 288.

Current subspecies: *C. senegalensis goliathula* Forel.

##### Material examined.

**KSA**: Asir province: Raydah: 18.204267N, 42.4124E, 2820 m, 21.ii.2014 (Al Dhafer et al.) (18 w, KSMA); Raydah: 18.221667N, 42.404167E, 2600 m, 13.iv.2011 (Sharaf MR) (21 w, 1 q, KSMA); Al Souda, 18.274167N, 42.364444E, 2982 m, 24.iv.2011 (Sharaf MR) (9 w, KSMA).

##### Geographic range.

While this species was originally described from Senegal, it seems to have a fairly disjunctive distribution since it is known from eastern and northwestern Africa without being recorded from countries in-between (Guénard et al. 2017; Janicki et al. 2017). In the Arabian Peninsula, it was recorded from the KSA, Oman, and Yemen (Collingwood 1985; Collingwood and Agosti 1996; Collingwood et al. 2011; Borowiec 2014; Sharaf et al. 2018) (Fig. 25).

##### Remarks.

The disjunct distribution of this species is a bit unusual and might require further attention in future studies of Afrotropical *Crematogaster*. It is likely that the odd distribution is just based on a sampling artifact or a preference of arid habitats, which are not as common in Central Africa. However, it could also be the case that this species, as currently defined, consists of several cryptic taxa. Without a thorough revision of the Afrotropical fauna it is impossible to be sure and we therefore list material examined as *C. senegalensis*.

**Figure 29.**
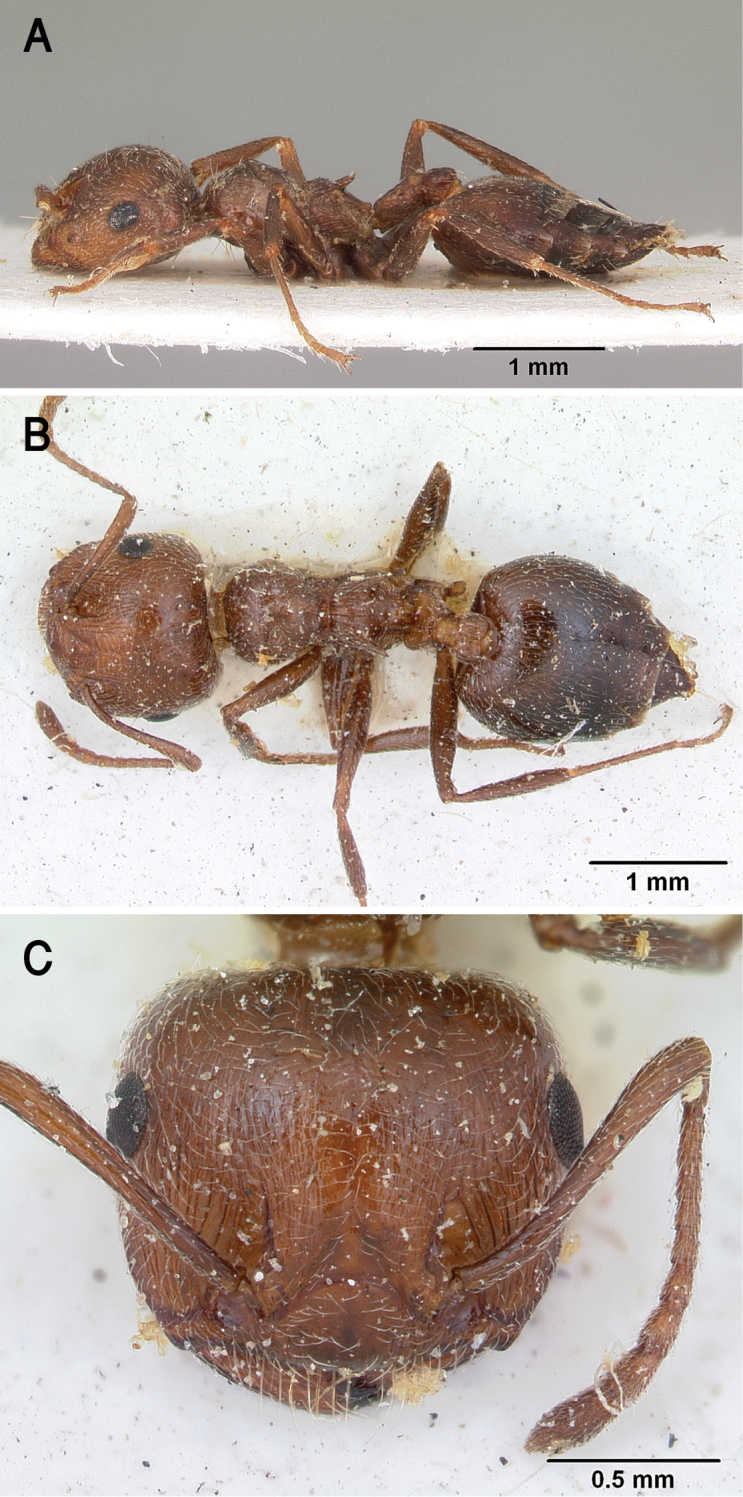
*C. senegalensis***A** body in profile **B** body in dorsal view **C** head in full-face view, CASENT0104592 (April Nobile), www.AntWeb.org.

#### Crematogaster striaticeps

Taxon classificationAnimaliaHymenopteraFormicidae

Forel stat. nov.

ED9A5D5B-2DA2-53F8-808A-521480D3F86D

Figure 30A–C

##### Taxonomic history.

*Crematogaster laestrygon striaticeps* Forel, 1909: 104 (w.) Algeria; Santschi 1937: 308 (q., m.).

Combination in Crematogaster (Acrocoelia): Emery 1922: 142.

##### Geographic range.

This species was described from Algeria and is also found in the neighboring countries of Tunisia and Lybia (Borowiec 2014; Guénard et al. 2017; Janicki et al. 2017). In the Arabian Peninsula, it has been recorded from the KSA (Collingwood 1985).

##### Remarks.

*Crematogaster striaticeps* was originally described as a subspecies of *C. laestrygon* but herein we treat this taxon as a good species. The main difference responsible for the decision to raise *striaticeps* to the specific rank is the presence of dense longitudinal striations on the entire cephalic surface whereas *laestrygon* has a smooth cephalic surface and longitudinal striations are feebly developed and restricted to the area in front of eyes.

We have been hesitant with this decision since both taxa are similar to other Afrotropical or Mediterranean *Crematogaster* taxa and it is not clear which taxonomic status they may assume after a thorough revision of the genus. However, since we established that *C. laestrygon* and *C. striaticeps* are not the same taxon, we propose to separate them by raising the latter to species rank to achieve clarity of their status for the Arabian region and future studies of its fauna.

**Figure 30.**
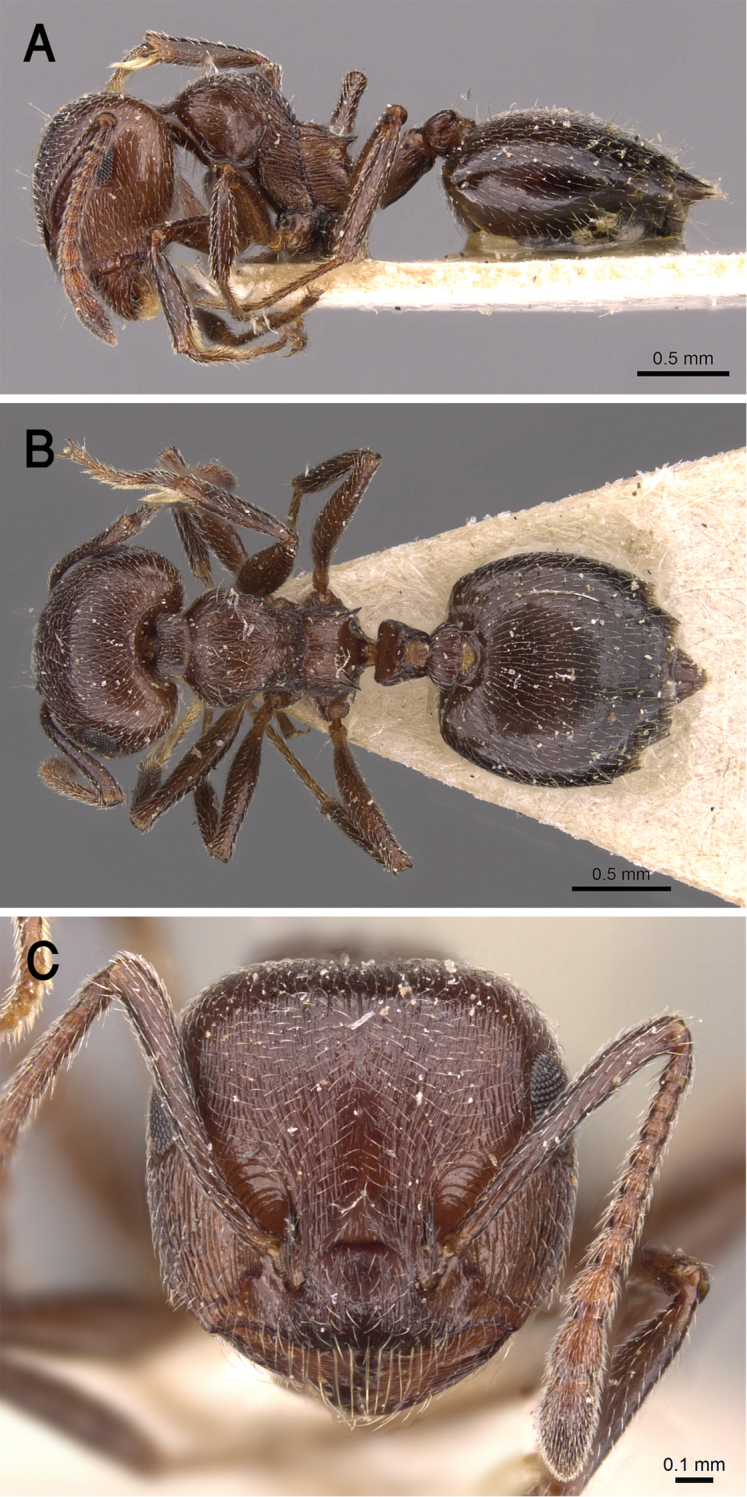
*C. striaticeps***A** body in profile **B** body in dorsal view **C** head in full-face view, CASENT0908479 (Zach Lieberman), www.AntWeb.org.

## Discussion

As can be seen from the species accounts presented above, the taxonomic histories of many species treated herein are complex and problematic. Many species have had numerous status changes and a changing number of infraspecific taxa. In some cases, it is likely that the species listed here will turn out to be senior or junior synonym of another taxon, and it is also very probable that some or many of the infraspecific taxa deserve to be treated as “good” species. As a consequence, except for the few species endemic to the Arabian Peninsula, for most others we suggest caution. Our review of species is based on literature records, material examined by the first author, and Arabian material examined in some European collections. We have pointed out which species we consider well identifiable and which ones are difficult. Overall, we consider previous identifications, as well as ours, as temporary. This study is meant as a first step stone towards a more comprehensive revision of the Arabian *Crematogaster* fauna. Since comprehensive taxonomic revisions of the genus are not to be expected from neither the Palaearctic nor the Afrotropical regions any time soon, the most sensible solution for the study of Arabian *Crematogaster* is to visit additional European collections and compare our material with as many types as possible in order to verify or improve the identifications of our material.

Notwithstanding the taxonomic problems lined out above, the treated fauna of *Crematogaster* exemplifies very well that the Arabian Peninsula shares substantial faunal elements with the Palaearctic (mostly Mediterranean species) and the Afrotropics, with a minority of species currently considered as Arabian endemics. At present, we recognize eight Afrotropical, seven Palaearctic, and two Arabian species, which we think fits fairly well with the biogeography of the Arabian Peninsula. However, this assessment might change with further studies and comparisons with types. We suspect that in some cases it might turn out that our material is not conspecific with any of the previously identified species and might represent another undescribed endemic, but this requires further taxonomic work.

The ant genus *Crematogaster* is one of the most abundant myrmicine genera in the Arabian Peninsula, especially in the Asir mountains, Yemen, and Oman, particularly in areas with open forests and woodlands of *Acacia* (Martius, 1829) (Fabaceae) and *Juniperus* L. (Cupressaceae) trees. The close ecological association between ants and *acacia* trees has been documented by several authors (e.g., Hölldobler and Wilson 1990; Isbell et al. 2013; Madden and Young 1992; Palmer and Brody 2013; Young et al. 1997). *Crematogaster mimosae* is known to have mutualistic relationships with other ants in East Africa (Boyle et al. 2017; Palmer and Brody 2013) and we anticipate similar associations to be found in the vast areas of *Acacia* forests of the southwestern part of the Arabian Peninsula.

Although at present only 17 species of *Crematogaster* are known from the Arabian Peninsula, further targeted collecting may yield both additional species records and more new species. For example, no *Crematogaster* have been collected from Bahrain but it is very unlikely that the genus is absent from the country. We are sure that the current absence of *Crematogaster* records from Bahrain is due to the lack of any national myrmecological studies. The identification key to the Arabian *Crematogaster* presented herein serves as a foundation for further faunistic studies and taxonomic revisions of the genus.

## Supplementary Material

XML Treatment for Crematogaster acaciae

XML Treatment for Crematogaster aegyptiaca

XML Treatment for Crematogaster antaris

XML Treatment for Crematogaster auberti

XML Treatment for Crematogaster chiarinii

XML Treatment for Crematogaster delagoensis

XML Treatment for Crematogaster flaviventris

XML Treatment for Crematogaster gryllsi

XML Treatment for Crematogaster inermis

XML Treatment for Crematogaster jacindae

XML Treatment for Crematogaster jehovae

XML Treatment for Crematogaster laestrygon

XML Treatment for Crematogaster melanogaster

XML Treatment for Crematogaster mimosae

XML Treatment for Crematogaster oasium

XML Treatment for Crematogaster senegalensis

XML Treatment for Crematogaster striaticeps
